# Carbohydrate fatty acid monosulphate: oil-in-water adjuvant enhances SARS-CoV-2 RBD nanoparticle-induced immunogenicity and protection in mice

**DOI:** 10.1038/s41541-023-00610-4

**Published:** 2023-02-14

**Authors:** Etsuro Nanishi, Francesco Borriello, Hyuk-Soo Seo, Timothy R. O’Meara, Marisa E. McGrath, Yoshine Saito, Jing Chen, Joann Diray-Arce, Kijun Song, Andrew Z. Xu, Soumik Barman, Manisha Menon, Danica Dong, Timothy M. Caradonna, Jared Feldman, Blake M. Hauser, Aaron G. Schmidt, Lindsey R. Baden, Robert K. Ernst, Carly Dillen, Jingyou Yu, Aiquan Chang, Luuk Hilgers, Peter Paul Platenburg, Sirano Dhe-Paganon, Dan H. Barouch, Al Ozonoff, Ivan Zanoni, Matthew B. Frieman, David J. Dowling, Ofer Levy

**Affiliations:** 1 Precision Vaccines Program, Boston Children’s Hospital, Boston, MA USA; 2 Department of Pediatrics, Harvard Medical School, Boston, MA USA; 3 Division of Immunology, Boston Children’s Hospital, Boston, MA USA; 4 Department of Cancer Biology, Dana-Farber Cancer Institute, Boston, MA USA; 5 Department of Biological Chemistry and Molecular Pharmacology, Harvard Medical School, Boston, MA USA; 6 Department of Microbiology and Immunology, Center for Pathogen Research, University of Maryland School of Medicine, Baltimore, MD USA; 7 Research Computing Group, Boston Children’s Hospital, Boston, MA USA; 8 Ragon Institute of MGH, MIT, and Harvard, Cambridge, MA USA; 9 Department of Microbiology, Harvard Medical School, Boston, MA USA; 10 Department of Medicine, Brigham and Women’s Hospital, Boston, MA USA; 11 Department of Microbial Pathogenesis, University of Maryland School of Dentistry, Baltimore, MD USA; 12 Center for Virology and Vaccine Research, Beth Israel Deaconess Medical Center, Harvard Medical School, Boston, MA USA; 13 LiteVax B.V, Oss, The Netherlands; 14 Broad Institute of MIT & Harvard, Cambridge, MA USA; 15 Present Address: Generate Biomedicines, Cambridge, MA USA

**Keywords:** Adjuvants, Antibodies

## Abstract

Development of SARS-CoV-2 vaccines that protect vulnerable populations is a public health priority. Here, we took a systematic and iterative approach by testing several adjuvants and SARS-CoV-2 antigens to identify a combination that elicits antibodies and protection in young and aged mice. While demonstrating superior immunogenicity to soluble receptor-binding domain (RBD), RBD displayed as a protein nanoparticle (RBD-NP) generated limited antibody responses. Comparison of multiple adjuvants including *AddaVax*, *AddaS03*, and AS01B in young and aged mice demonstrated that an oil-in-water emulsion containing carbohydrate fatty acid monosulphate derivative (CMS:O/W) most effectively enhanced RBD-NP-induced cross-neutralizing antibodies and protection across age groups. CMS:O/W enhanced antigen retention in the draining lymph node, induced injection site, and lymph node cytokines, with CMS inducing MyD88-dependent Th1 cytokine polarization. Furthermore, CMS and O/W synergistically induced chemokine production from human PBMCs. Overall, CMS:O/W adjuvant may enhance immunogenicity and protection of vulnerable populations against SARS-CoV-2 and other infectious pathogens.

## Introduction

The SARS-CoV-2 pandemic is a global health crisis necessitating widespread deployment of effective vaccines across all ages. Basic research into prototype betacoronavirus pathogens coupled with advances in structure-based antigen design, protein engineering, and new manufacturing platforms, such as mRNA and adenoviral virus vectors have enabled the development of effective vaccines at an unprecedented speed^1,2^. Nevertheless, limitations to the current mRNA vaccines include need for controlling the spread of the virus worldwide and especially in low- and middle-income countries will likely require global deployment of safe, effective, affordable, scalable, and practical vaccines that can also protect highly vulnerable populations including older adults individuals against COVID-19-related morbidity and mortality^3–6^. Protein subunit vaccines may meet at least some of these criteria and offer additional advantages of not requiring ultra-cold storage and having a long track-record of safety. Indeed, SARS-CoV-2 subunit vaccines have already shown promising results in pre-clinical and clinical studies^7–15^.

Most vaccines currently in use or clinical development target the SARS-CoV-2 Spike glycoprotein due to the key role of its receptor-binding domain (RBD) in binding to the human receptor angiotensin-converting enzyme 2 (ACE2) and mediating cell entry^16–18^. The RBD protein would be an ideal candidate for a subunit vaccine since it is targeted by neutralizing antibodies (Abs) that exert a protective role against SARS-CoV-2 infection, and it is readily produced at scale^13,19–23^. However, RBD is poorly immunogenic, limiting its use as a candidate vaccine antigen. Structure-based antigen design can enhance immunogenicity of vaccines^24–30^. Indeed, RBD antigens have been generated as dimers, trimers, or displayed onto protein or synthetic nanoparticles with the goal of increasing antigen trafficking to the draining lymph node (dLN) and/or promoting clustering and activation of the B cell receptor^13,15,31–41^. While promising, structurally optimized RBD antigen alone induces suboptimal immunogenicity^42^.

Thus, the addition of adjuvants, vaccine components that enhance antigen immunogenicity by activating innate immunity and/or modulating antigen pharmacokinetics^43–46^ represents a promising approach. For example, adjuvantation with AS03 improved RBD immunogenicity and demonstrated superior neutralizing Ab response as compared to currently authorized ChAdOx1 nCoV-19 vaccine in clinical trials^42,47^. Although a growing number of vaccine adjuvants has been developed, most studies only evaluate 1 or 2 adjuvants^48^. We, therefore, expanded the range enabling direct comparison of multiple adjuvants. Adjuvantation of candidate RBD antigens and evaluation in pre-clinical models that take into account age-dependent vaccine immune responses and COVID-19 susceptibility may enable down-selecting and prioritizing adjuvanted RBD antigen-based vaccines^49^.

Here, we studied adjuvantation of a nanoparticle comprised of multimeric RBD displayed onto a protein scaffold composed of 60 subunits of the self-assembling bacterial protein lumazine synthase^50^. The RBD nanoparticle (RBD-NP) demonstrated greater immunogenicity than pre-fusion stabilized Spike trimer^51^ (hereafter Spike) or monomeric RBD, but this was still limited as indicated by low levels of Ab and T cell responses, prompting an effort to identify a robust adjuvantation system. Directly comparing multiple adjuvant formulations, including *AddaVax*, *AddaS03*, and AS01B, in combination with RBD-NP, we found that a squalane-based oil-in-water (O/W) emulsion containing synthetic Carbohydrate fatty acid MonoSulphate derivative (CMS)^52^ further enhanced anti-RBD serum Ab titers and SARS-CoV-2 cross-neutralizing titers in both young and aged mice. Mechanistically, CMS:O/W induced antigen retention in the dLN and expression of pro-inflammatory cytokines at the injection sites and type I interferon (IFN)-dependent IFN stimulated genes (ISGs) in the dLN. We further demonstrated that CMS contributes to Th1 polarization via proinflammatory cytokine production dependent on MyD88, a TLR adaptor protein that mediates several proinflammatory signaling pathways. Overall, our study demonstrates the utility of a systematic approach to develop and optimize an adjuvanted RBD-NP effective across multiple age groups. Our results may inform a practical and scalable approach to develop adjuvanted subunit vaccines against SARS-CoV-2 and other viral pathogens tailored for vulnerable older adults.

## Results

### In vitro characterization of RBD-NP reveals high-density display of multimeric RBD

**Fig. 1:**
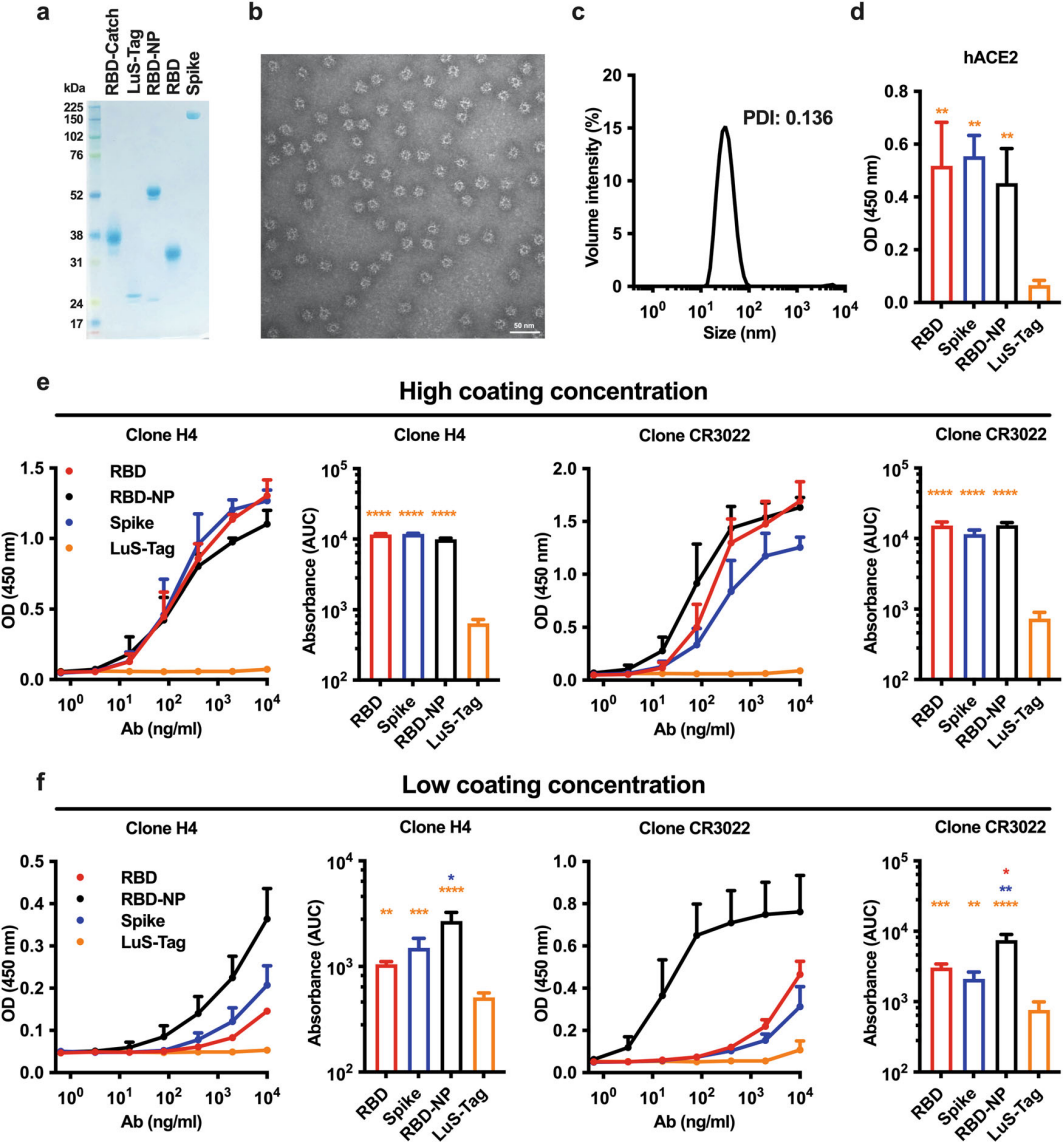
A lumazine synthase nanoparticle scaffold enables efficient RBD display. **a** SDS-PAGE analysis under reducing conditions of RBD expressing SpyCatcher (RBD-Catch), lumazine synthase expressing SpyTag (LuS-Tag), RBD nanoparticle (RBD-NP), as well as native RBD and Spike proteins. **b**, **c** Transmission electron microscopy (**b**) and dynamic light scattering (**c**) analyses of RBD-NP. Scale bar, 50 nm. **d**–**f** ELISA plates were coated with RBD, Spike, RBD-NP and LuS-Tag at 1 μg/ml (**d**), 5 μg/ml (**e**) or 0.5 μg/ml (**f**). Binding of recombinant human ACE2 (hACE2) or anti-RBD H4 and CR3022 Ab clones tested at multiple concentrations was expressed as optical density (OD) at 450 nm or area under the curve (AUC). Data are presented as mean and SD. *N* = 3–6 experiments. *, **, ***, and **** respectively indicate *p* < 0.05, 0.01, 0.001, and 0.0001. Statistical significance was determined by one-way ANOVA corrected for multiple comparisons. The color code indicates comparisons among experimental groups. PDI: polydispersity index. **c–e** Source data are provided as Supplementary Data.

High-density display of antigens onto protein NPs increases their immunogenicity and has been employed in several vaccine candidates against viral infections to elicit robust serum antigen-specific Ab titers^25^. In order to assemble SARS-CoV-2 RBD onto a protein NP scaffold, we took advantage of the SpyTag/SpyCatcher conjugation system in which proteins fused with SpyTag and SpyCatcher spontaneously form stable isopeptide bonds^53^. Briefly, we used the self-assembling lumazine synthase (LuS) from the hyperthermophile “*Aquifex aeolicus”* as protein NP scaffold^50^ and respectively expressed RBD and LuS with SpyCatcher (RBD-Catch) and SpyTag (LuS-Tag). SDS-PAGE analysis under reducing conditions of RBD-Catch, LuS-Tag, RBD-NP (generated by conjugating RBD-Catch and LuS-Tag) as well as native RBD and Spike proteins confirmed expected molecular weights (Fig. 1a). SDS-PAGE analysis demonstrated multiple bands of RBD-NP (Fig. 1a), which was likely due to heterogeneous glycosylation patterns since doublets were no longer observed following Peptide -N-Glycosidase F (PNGase F) treatment (Supplementary Fig. 1). Transmission electron microscopy analysis of RBD-NP revealed a ruffled border, suggesting efficient conjugation and display of RBD onto the protein scaffold and homogeneous size (Fig. 1b). This impression was further confirmed by dynamic light scattering analysis, showing an average size of ~30 nm (Fig. 1c). To confirm the proper display of RBD onto NPs, we coated ELISA plates with RBD, Spike, RBD-NP, LuS-Tag, and assessed binding to recombinant human ACE2 (hACE2) and two anti-RBD monoclonal Abs (mAbs: clones H4 and CR3022). RBD-NP binding to hACE2, clones H4 and CR3022 were comparable to RBD and Spike, while no binding to LuS-Tag was observed (Fig. 1d, e). RBD-NP binding profiles remained unaltered under multiple storage conditions, namely five freeze/thaw cycles or storage for 1 week at 4 °C or room temperature (Supplementary Fig. 2). Interestingly, by assessing binding to mAb clones H4 and CR3022 under lower coating concentrations of RBD, Spike, and RBD-NP, we observed preferential binding to RBD-NP (Fig. 1f), suggesting that high-density display of RBD onto NP increases Ab avidity. To explore another functional correlate of RBD-NP structure, we performed a competition assay in which Vero cells were incubated with SARS-CoV-2 in the absence or presence of multiple concentrations of RBD, Spike, or RBD-NP. RBD-NP significantly reduced SARS-CoV-2 infection as assessed by IC50 and AUC (Supplementary Fig. 3), further supporting the high-density display of RBD onto NP.

### Immunization with RBD-NP elicits high serum anti-RBD antibody titers and SARS-CoV-2 neutralizing titers

**Fig. 2:**
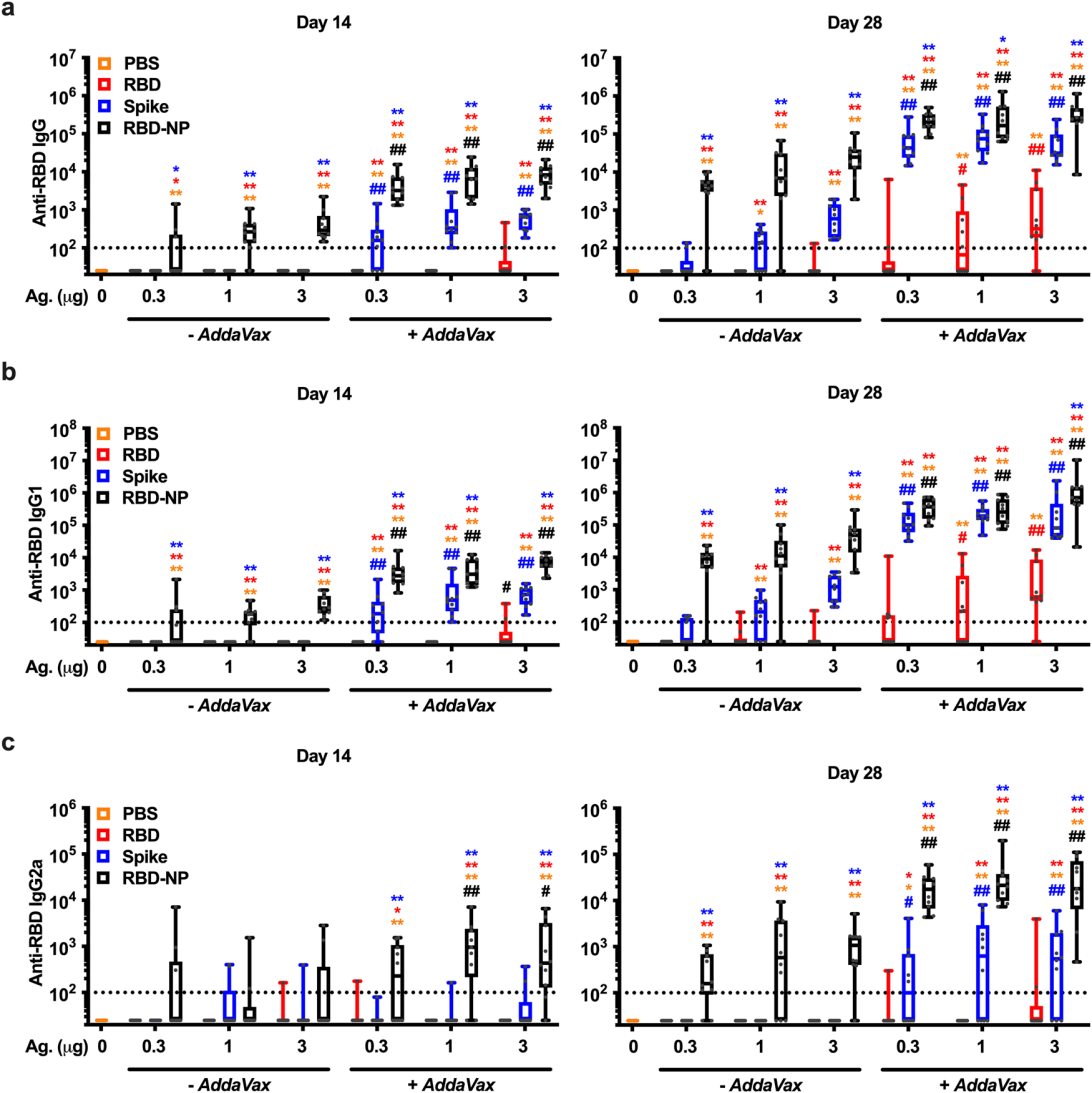
RBD nanoparticle demonstrates superior immunogenicity to Spike or monomeric RBD in mice. 3-month-old BALB/c mice were injected with PBS or immunized with the indicated doses (0.3, 1, or 3 µg) of RBD, Spike, or RBD nanoparticle (RBD-NP) alone or formulated with *AddaVax* on Day 0 (prime) and 14 (boost). Anti-RBD IgG (**a**), IgG1 (**b**), and IgG2a (**c**) antibody titers were assessed in serum samples collected on Days 14 (pre-boost) and 28. Dotted lines indicate a lower limit of detection. *N* = 7–10 mice per group. * and ** respectively indicate *p* < 0.05 and 0.01 for comparisons among RBD, Spike, and RBD-NP in the same adjuvant formulation group (- *AddaVax* or + *AddaVax*). # and ## respectively indicate *p* < 0.05 and 0.01 for comparisons of the same antigen groups between the two adjuvant formulation groups (- *AddaVax* vs. + *AddaVax*). Statistical significance was determined by two-way ANOVA corrected for multiple comparisons after Log-transformation of the raw data. The color code indicates comparisons among experimental groups. Box-and-whisker plots represent the minimum, first quartile, median, third quartile, and maximum value. Each symbol represents an individual mouse. Source data are provided as Supplementary Data.

**Fig. 3:**
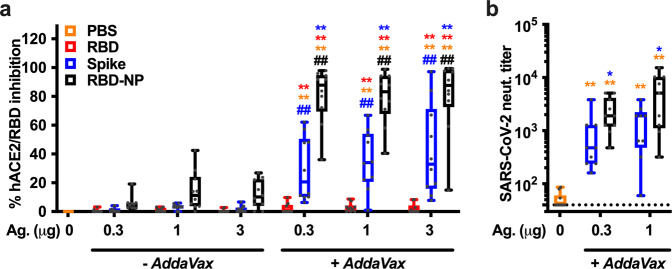
Immunization with RBD nanoparticles induce robust SARS-CoV-2 neutralizing titers in mice at all doses tested. 3-month-old BALB/c mice were injected with PBS or immunized with the indicated doses (0.3, 1, or 3 µg) of RBD, Spike, or RBD nanoparticle (RBD-NP) alone or formulated with *AddaVax* on Day 0 (prime) and 14 (boost). Serum levels of anti-RBD neutralizing antibodies were assessed on Day 28 by SARS-CoV-2 surrogate (**a**) and conventional (**b**) virus neutralization tests. The dotted line indicates a lower limit of detection. *N* = 7–10 mice per group. * and ** respectively indicate *p* < 0.05 and 0.01 for comparisons among RBD, Spike, and RBD-NP in the same adjuvant formulation group (− *AddaVax* or + *AddaVax*). ## indicates *p* < 0.01 for comparisons of the same antigen groups between the two adjuvant formulation groups (− *AddaVax* vs. + *AddaVax*). Statistical significance was determined by two-way ANOVA corrected for multiple comparisons. Data shown in **b** were Log-transformed before the analysis. The color code indicates comparisons among experimental groups. Box-and-whisker plots represent the minimum, first quartile, median, third quartile, and maximum value. Each symbol represents an individual mouse. Source data are provided as Supplementary Data.

To assess whether RBD-NP increases RBD immunogenicity, we immunized BALB/c mice with multiple doses (namely, 0.3, 1, and 3 µg per mouse) of RBD, Spike, or RBD-NP alone or formulated with the MF59-like O/W emulsion *AddaVax* using a prime (Day 0) - boost (Day 14) schedule (Fig. 2). As expected, formulation with *AddaVax* enhanced anti-RBD Ab titers as compared to immunizations with non-adjuvanted antigens. In all experimental conditions, RBD-NP induced the highest titers of anti-RBD IgG, IgG1, and IgG2a, especially at the lowest tested dose (0.3 μg), thus showing a robust dose-sparing effect. Of note, anti-RBD Abs elicited by immunization with RBD-NP also recognized native RBD on Spike (Supplementary Fig. 4), which is key for SARS-CoV-2 neutralization. To confirm this point, we performed a surrogate of virus neutralization test (sVNT) that measures the degree of inhibition of RBD binding to hACE2 by immune sera, as well as a neutralization assay with live SARS-CoV-2 virus. In both assays, immunization with RBD-NP formulated with *AddaVax* induced higher levels of neutralization compared to immunization with Spike (Fig. 3a, b), while immunization with monomeric RBD failed to elicit significant levels of neutralization in the sVNT (Fig. 3a). Of note, immunization with RBD-NP formulated with *AddaVax* elicited high levels of anti-RBD neutralizing Abs in one additional inbred (C57BL/6) and one outbred (CD-1) mouse strains (Supplementary Fig. 5). Overall, these results show that multimeric RBD displayed on a NP significantly enhances its immunogenicity across multiple mouse strains, eliciting high anti-RBD neutralizing Abs with a significant dose-sparing effect.

### CMS:O/W adjuvant significantly enhances RBD-NP immunogenicity in young and aged mice

**Fig. 4:**
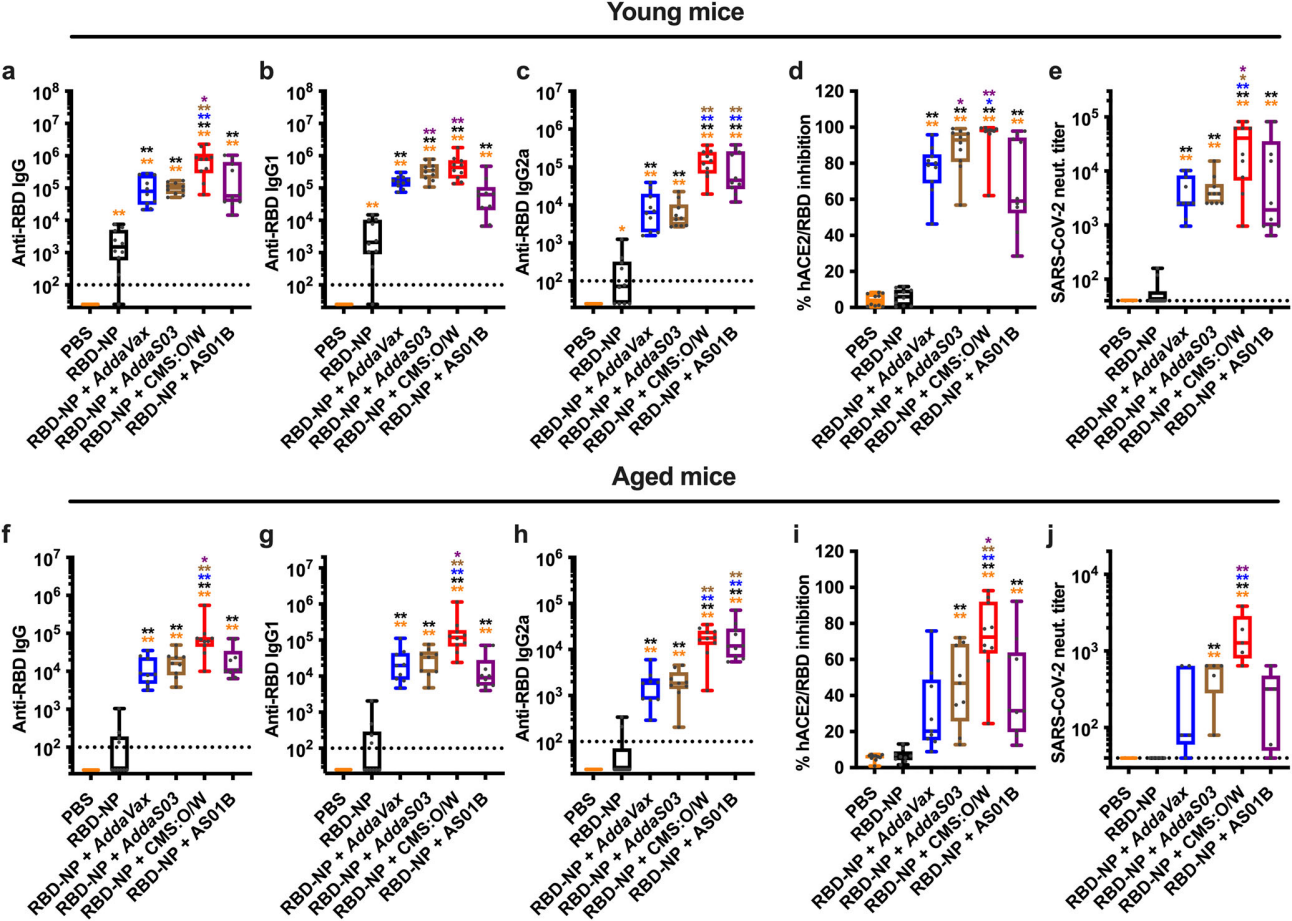
CMS:O/W adjuvant enhances RBD nanoparticle immunogenicity in young and aged mice. Young (3-month-old, **a**–**e**) and aged (14-month-old, **f**–**j**) BALB/c mice were immunized on Day 0 (prime) and 14 (boost) with PBS, 0.3 µg of RBD nanoparticle (RBD-NP) alone or formulated with *AddaVax*, *AddaS03*, CMS:O/W adjuvant or AS01B. Serum samples were collected on Day 28 to assess anti-RBD IgG (**a**, **f**), IgG1 (**b**, **g**), IgG2a (**c**, **h**) antibody titers as well as anti-RBD neutralizing activity by surrogate (**d**, **i**) and conventional (**e**, **j**) virus neutralization tests. Dotted lines indicate lower limit of detection. *N* = 10 (**a**–**i**) or 5 (**j**) mice per group. * and ** respectively indicate *p* < 0.05 and 0.01. Statistical significance was determined by one-way ANOVA corrected for multiple comparisons. Data shown in (**a**–**c**, **e**, **f**–**h**, **j**) w**e**re Log-transformed before the analysis. The color code indicates comparisons among experimental groups. Box-and-whisker plots represent the minimum, first quartile, median, third quartile, and maximum value. Each symbol represents an individual mouse. Source data are provided as Supplementary Data.

Adjuvants play a key role in enhancing antigen immunogenicity^43–46^. Although it is possible to generalize desirable properties for an adjuvant, a proper match between an adjuvant and a specific antigen has to be empirically evaluated. For example, comparisons of O/W- and aluminum hydroxide-based adjuvant formulations with RBD-NP demonstrated that the alpha-tocopherol-containing squalene-based oil-in-water emulsion AS03 is particularly effective at enhancing RBD-NP immunogenicity in non-human primates^36^. We, therefore, evaluated the immunogenicity of our RBD-NP with several O/W emulsions, namely *AddaVax*, the AS03-like adjuvant *AddaS03*, and a squalane-based O/W emulsion containing Carbohydrate fatty acid MonoSulphate derivative (CMS)^52^. As a key benchmark, we also included AS01B (a liposome-based adjuvant containing monophosphoryl lipid A and saponin QS-21) as a clinical-grade benchmark adjuvant with potent immunostimulatory activity^54,55^. As an experimental model, we chose to immunize both young (3-month-old) and aged (14-month-old) mice to assess whether an optimized vaccine formulation could overcome impaired vaccine immunogenicity associated with immunosenescence in aged populations^56^. All adjuvanted RBD-NP vaccine formulations induced robust titers of anti-RBD neutralizing Abs in young mice (Fig. 4a–e). Of note, CMS:O/W-adjuvanted RBD-NP vaccine elicited the highest levels of anti-RBD IgG Abs (Fig. 4a) by enhancing both anti-RBD IgG and IgG2a titers (Fig. 4b, c), resulting in potent inhibition of RBD binding to hACE2 (Fig. 4d) and SARS-CoV-2 neutralization (Fig. 4e). Immunization of aged mice resulted in overall lower anti-RBD Ab titers compared to young mice (Supplementary Fig. 6). Although we attribute the lower Ab titers to aging, we cannot exclude an effect of genetic or environmental differences as the young and aged BALB/c mice were purchased from different breeding colonies. Nevertheless, CMS:O/W again induced the highest anti-RBD Ab titers (Fig. 4f–h), inhibition of RBD binding to hACE2 (Fig. 4i), and SARS-CoV-2 neutralization (Fig. 4j) in aged mice.

### Immunization with RBD-NP formulated with CMS:O/W adjuvant protects aged mice from SARS-CoV-2 challenge and elicits cross-neutralizing antibodies

**Fig. 5:**
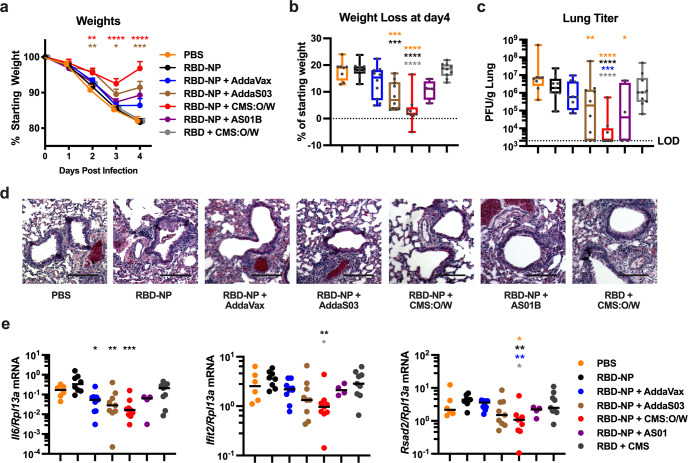
Immunization with CMS:O/W-adjuvanted RBD nanoparticle completely protects aged mice from SARS-CoV-2 challenge. Aged (14-month-old) BALB/c mice were immunized on Day 0 (prime) and 14 (boost) with PBS, 0.3 µg of RBD nanoparticle (RBD-NP) alone or formulated with *AddaVax*, *AddaS03*, CMS:O/W, or AS01B, or 10 µg of monomeric RBD formulated with CMS:O/W. Seven weeks after the final immunization, mice were infected with 10^3^ plaque-forming units (PFU) of mouse-adapted SARS-CoV-2 and monitored for up to 4 days for weight loss. Daily weights (**a**) and weight loss at 4 days post infection (**b**) of infected mice are shown. **c**–**e** On Day 4, mice were euthanized, and lungs were collected to assess viral titers (**c**), hematoxylin and eosin-stained lung images (**d**), and gene expression profiles shown as relative expression compared to *Rlp13a* (**e**). *N* = 8–10 per group except for AS01B group (*N* = 4). **a** Results represent mean ± SEM. Data were compared to the PBS group by Kruskal–Wallis test corrected for multiple comparisons. **b**, **c**, **e** Data were analyzed by one-way ANOVAs corrected for multiple comparisons. Asterisks indicate comparisons to groups indicated by the color code. **d** Representative lung images are shown. Scale bar, 500 µm. Box-and-whisker plots represent the minimum, first quartile, median, third quartile, and maximum value. Each symbol represents an individual sample. *, **, ***, and **** respectively indicate *p* < 0.05, 0.01, 0.001, and 0.0001. Source data are provided as Supplementary Data.

Neutralizing Ab titers are an important correlate of protection against SARS-CoV-2 infection^57–59^. We next assessed whether the high titers of neutralizing Abs in mice immunized with CMS:O/W-adjuvanted RBD-NP translate into enhanced protection against SARS-CoV-2 infection over other adjuvants. To this end, we immunized aged BALBc mice with 0.3 µg of RBD-NP formulated with CMS:O/W, *AddaVax*, *AddaS03*, or AS01B and challenged them with 10^3^ PFU of the mouse-adapted strain SARS-CoV-2 MA10^60^ (Fig. 5). Mice immunized with 10 µg of RBD formulated with CMS:O/W were also included to assess relative protection induced by RBD-NP as compared to monomeric RBD. Strikingly, aged mice immunized with RBD-NP formulated CMS:O/W were protected from weight loss, whereas mice receiving non-adjuvanted RBD-NP or CMS:O/W-adjuvanted monomeric RBD showed significant weight loss comparable to the naive mice (Fig. 5a, b). Mice immunized with RBD-NP formulated with a benchmarking adjuvant, namely *AddaVax*, *AddaS03*, or AS01B, were partially protected. Lung viral titers (Fig. 5c), lung histopathological analysis (Fig. 5d), and gene expression of type I interferon signaling (*Ifit* and *Rsad2*) and *Il6* as drivers of pathological pulmonary responses after SARS-CoV-2 infection^61^ (Fig. 5e) further confirmed the reduced SARS-CoV-2 infection in aged mice immunized with RBD-NP formulated with CMS:O/W adjuvant.

**Fig. 6:**
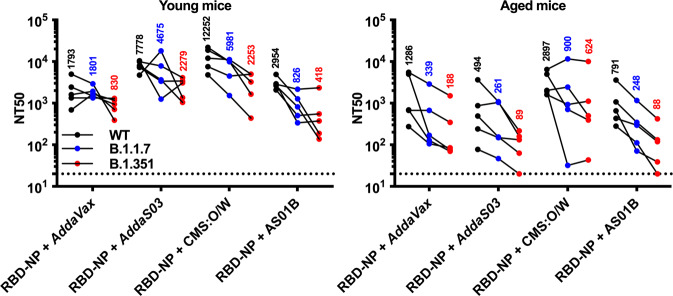
Immunization with CMS-adjuvanted RBD nanoparticles induces cross-neutralizing antibodies. Young (3-month-old) and aged (14-month-old) BALB/c mice were immunized on Day 0 (prime) and 14 (boost) with 0.3 µg of RBD nanoparticle (RBD-NP) formulated with *AddaVax*, *AddaS03*, CMS adjuvant or AS01B. Serum samples were collected on Day 28 to assess neutralizing titers (NT50) against SARS-CoV-2 wild type (WT), B.1.1.7 or B.1.351 pseudoviruses. Dotted lines indicate a lower limit of detection. *N* = 5 mice per group and each dot represent an individual sample. Numbers indicate geometric mean titers for each experimental group. Source data are provided as Supplementary Data.

Since the beginning of the pandemic, several SARS-CoV-2 variants have emerged and showed reduced neutralization by serum samples of convalescent or vaccinated subjects^62–64^. Therefore, we assessed neutralization of SARS-CoV-2 wild type (WT), B.1.1.7, and B.1.351 pseudoviruses by serum samples collected from young and aged mice immunized with 0.3 µg of RBD-NP formulated with *AddaVax*, *AddaS03*, CMS:O/W or AS01B (Fig. 6). As expected, we observed lower neutralizing titers against B.1.351 compared to WT. However, mice immunized with RBD-NP formulated with CMS:O/W showed the highest geometric mean titers against all SARS-CoV-2 variants, especially in aged mice.

### CMS:O/W and AS01B RBD-NP enhance CD4^+^ T cell responses in young and aged mice

**Fig. 7:**
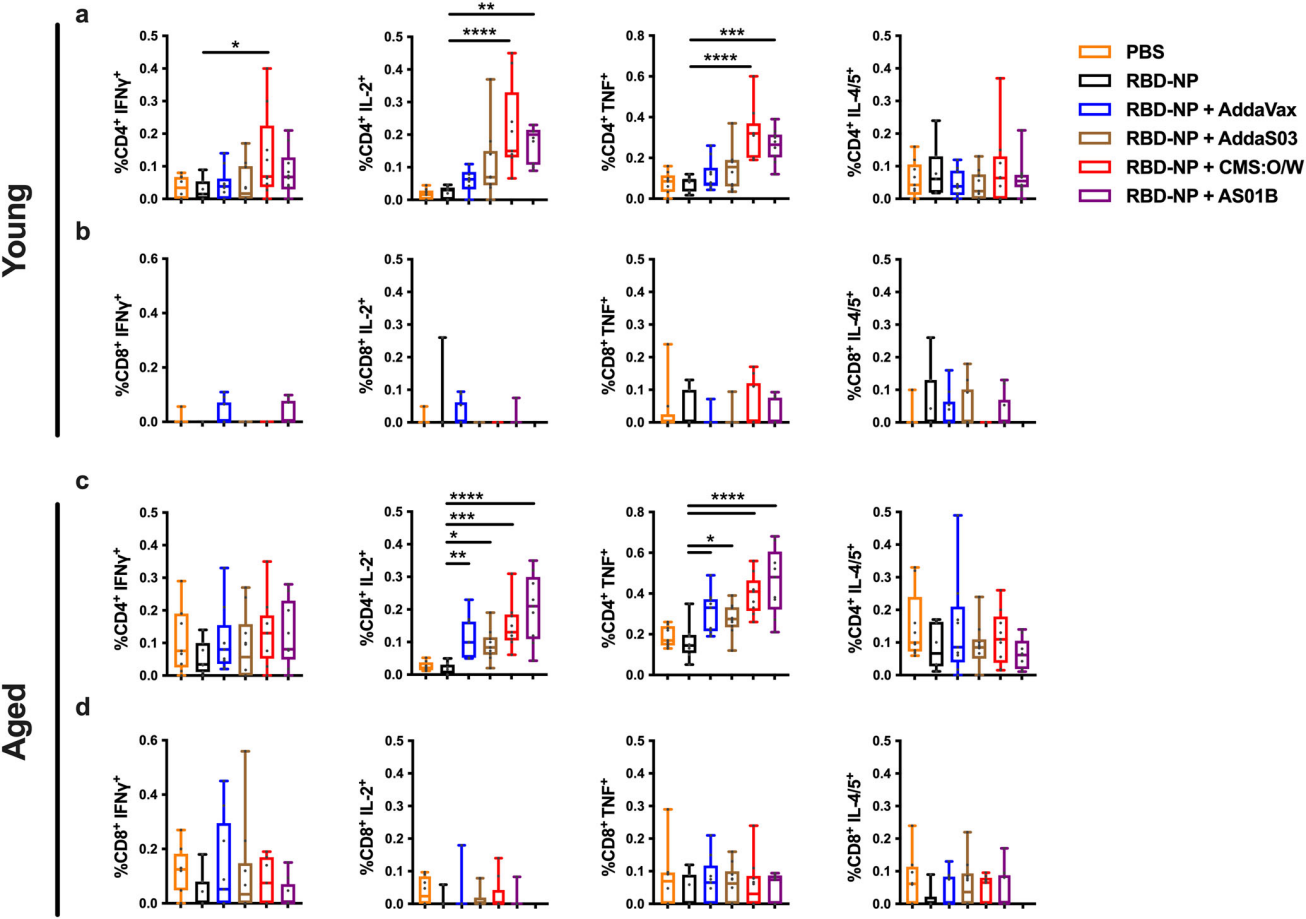
Adjuvanted RBD-NP enhances CD4+ T cell responses in young and aged mice. Young adult and aged BALB/c mice (3- and 11-month-old, respectively) were immunized on Day 0 (prime) and 14 (boost) with PBS, 0.3 µg of RBD nanoparticle (RBD-NP) alone or RBD-NP formulated with *AddaVax*, *AddaS03*, CMS:O/W adjuvant or AS01B. Splenocytes were collected 4 weeks after the final immunization and stimulated with a SARS-CoV-2 spike RBD peptide pool and expression of intracellular interferon-γ (IFNγ), TNF, IL-2, and IL-4/5 among CD4^+^ (**a**, **c**) and CD8^+^ (**b**, **d**) T cells were determined by flow cytometry. *N* = 8–10 per group. Data were log-transformed and analyzed by one-way ANOVAs corrected for multiple comparisons. *, **, *** and **** respectively indicate *p* < 0.05, 0.01, 0.001, and 0.0001. Box-and-whisker plots represent the minimum, first quartile, median, third quartile, and maximum value. Each symbol represents an individual sample. Source data are provided as Supplementary Data.

T cell responses induced by SARS-CoV-2 vaccines suppress viral replication and modulate disease severity^57,58,65^. We, therefore, analyzed specific T cell responses by stimulating splenocytes derived from immunized young and aged mice with a SARS-CoV-2 spike RBD peptide pool (Supplementary Fig. 7). CMS:O/W-adjuvanted RBD-NP demonstrated greater expression of interferon-γ (IFNγ), TNF, and IL-2 over non-adjuvanted RBD-NP among CD4^+^ T cells in young mice while *AddaVax*- and *AddaS03*-adjuvanted RBD-NP vaccines did not (Fig. 7a). Among aged mice, CMS:O/W- and AS01B-adjuvanted RBD-NP both elicited high TNF and IL-2 responses in CD4^+^ T cells, while IFNγ response was generally suppressed in this population (Fig. 7c). Contrary to CD4^+^ T cells, no significant CD8^+^ T cell responses were observed across treatment and age groups (Fig. 7b, d).

### CMS:O/W adjuvant promotes antigen retention in the draining lymph node

**Fig. 8:**
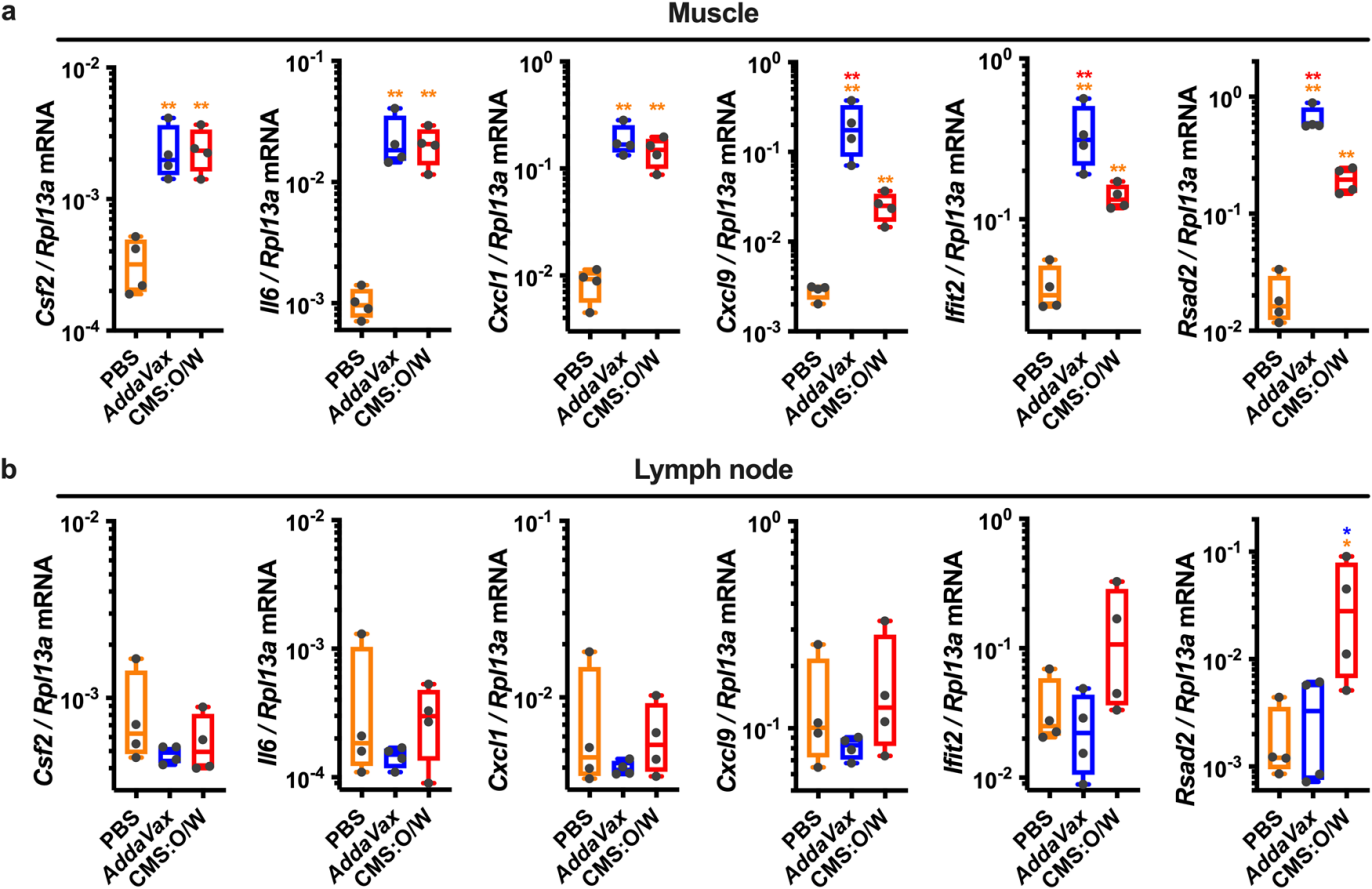
CMS:O/W adjuvant induces distinct gene expression profiles from *AddaVax*, including enhanced type I IFN-dependent ISG expression in the draining lymph node. **a**, **b** Young (3-month-old) BALB/c mice were injected with PBS, *AddaVax* and CMS:O/W adjuvant. 24 h later muscle tissue at the injection sites (**a**) and dLNs (**b**) were collected to assess gene expression profiles by qPCR. Results are reported as relative expression compared to *Rp13a*. *N* = 4 mice per group. * and ** respectively indicate *p* < 0.05 and 0.01. Statistical significance was determined by one-way ANOVA corrected for multiple comparisons after Log-transformation of the raw data. Box-and-whisker plots represent the minimum, first quartile, median, third quartile, and maximum value. Each symbol represents an individual sample. The color code indicates comparisons among experimental groups. Source data are provided as Supplementary Data.

O/W emulsions are highly effective adjuvants and act through multiple mechanisms, including (1) induction of a pro-inflammatory milieu at the injection site^66^ and/or (2) antigen targeting to and retention in the dLN^67^. We, therefore, assessed whether the enhanced adjuvanticity of CMS:O/W could be explained by any of these two mechanisms. To this end, we injected mice with R-Phycoerythrin (R-PE) as a model protein antigen with intrinsic fluorescence, alone or formulated with CMS:O/W or *AddaVax* that we used as benchmark adjuvant. Twenty-four hours post-injection, both *AddaVax* and CMS:O/W promoted significant and comparable antigen retention in the dLN (Supplementary Fig. 8). Interestingly, both adjuvants induced high gene expression of pro-inflammatory cytokines (*Csf2*, *Il6*, *Cxcl1*) and ISGs (*Cxcl9*, *Ifit2*, *Rsad2*) at the injection site, with *AddaVax* further enhancing the expression of the latter (Fig. 8a). However, only CMS:O/W enhanced type I IFN-dependent ISG expression in the dLN (Fig. 8b). Serum cytokine and chemokine concentrations were measured as a metric for systemic reactogenicity. Significant production of multiple cytokines and chemokines was observed in the serum of mice treated with AS01B, which is known for an acceptable safety profile in humans^44,68^ but not in CMS:O/W treated mice (Supplementary Fig. 9).

### CMS induces robust Th1-polarizing cytokine and chemokine production from human PBMCs and anti-RBD IgG2a Abs in mice

**Fig. 9:**
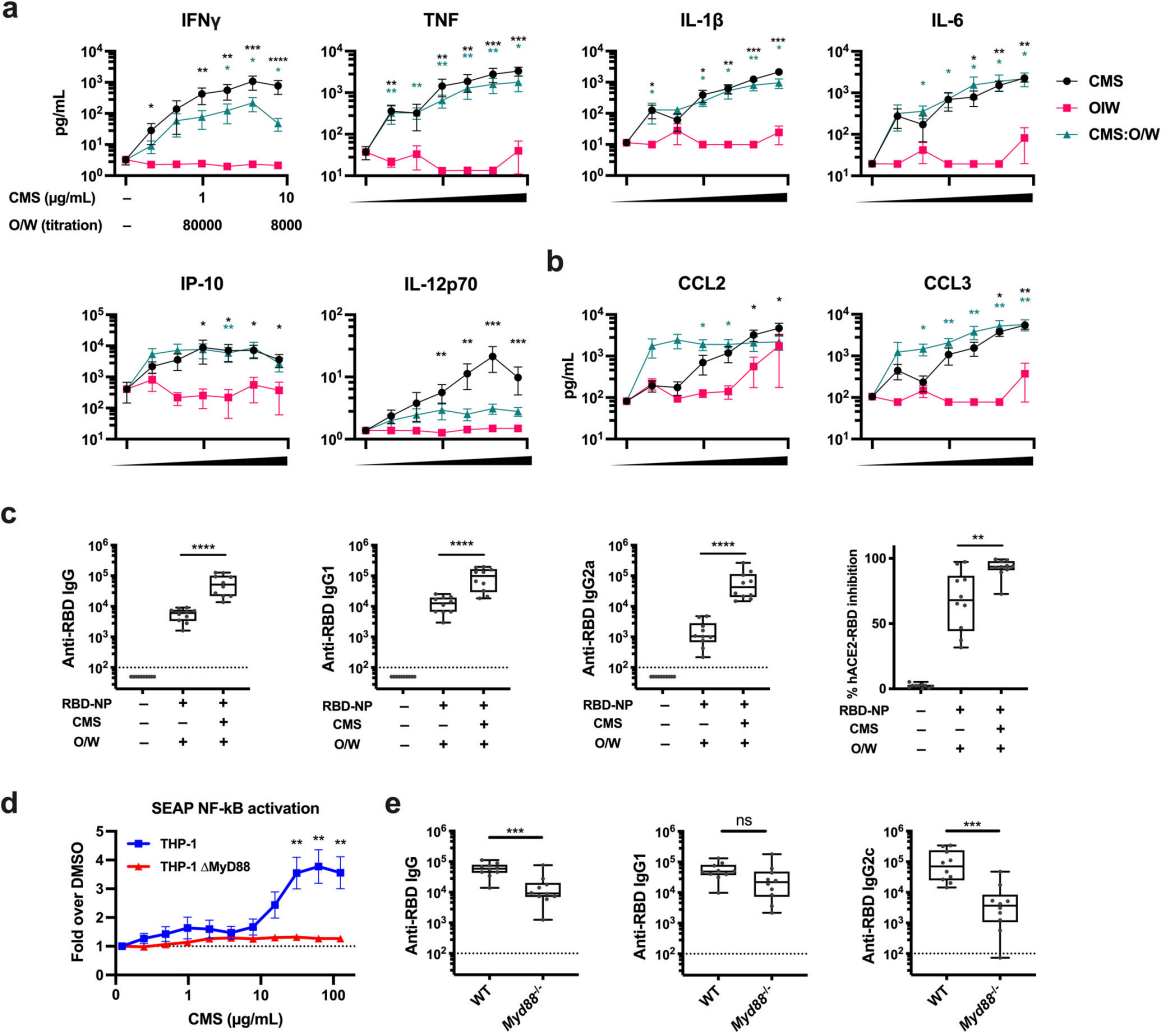
CMS induces MyD88-dependent cytokine production from human mononuclear cells in vitro and enhancement of murine anti-RBD IgG2a Ab production in vivo. **a**, **b** Human PBMCs were cultured in vitro for 24 h with CMS, O/W, or CMS:O/W formulation. Supernatants were collected for multiplexing bead array (**a**) or ELISA (**b**). Results are presented as mean ± SEM. *N* = 8. Black and green colored asterisks indicate comparisons of O/W to CMS and CMS:O/W groups, respectively. **c** Young (3-month-old) BALB/c mice were immunized on days 0 and 14 with RBD-NP with O/W alone or CMS:O/W formulation. Serum samples were collected on day 28. *N* = 10 mice per group. **d** A THP-1 human monocytic cell line stably expressing a secreted embryonic alkaline phosphatase (SEAP) reporter inducible by NF-κB was treated for 24 h with CMS at the indicated concentration. The amount of SEAP released into culture supernatants was quantified as a measure of NF-kB activation. Results of THP-1 and THP-1 MyD88 KO cells are presented as mean ± SEM. *N* = 8. **e** Young, 8-week-old C57BL6 WT or *Myd88*^−/−^ mice were immunized on days 0 and 14 with RBD-NP with CMS:O/W formulation. Serum samples were collected on day 28. *N* = 10 mice per group. Statistical significance was determined by either Mann–Whitney *U* test (**a**, **b**, **d**) or *t*-test (**c**, **e**). *, **, *** and **** respectively indicate *p* < 0.05, 0.01, 0.001 and 0.0001. Dotted lines indicate a lower limit of detection. Box-and-whisker plots represent the minimum, first quartile, median, third quartile, and maximum value. Each symbol represents an individual sample. Source data are provided as Supplementary Data.

To assess the translational relevance of our findings and dissect the mechanism of action of CMS:O/W adjuvant, we stimulated human peripheral blood mononuclear cells (PBMCs) with the individual components of CMS:O/W adjuvant, namely CMS and O/W, and compared them to the complete CMS:O/W formulation (Fig. 9a, b). Whereas O/W induced limited or no cytokine production, CMS robustly activated human leukocytes in a concentration-dependent manner and induced production of Th1-polarizing cytokines (e.g., IFNγ, TNF, IL-12) (Fig. 9a). Further, synergistic induction of CCL2 expression by the CMS:O/W formulation, as measured by Loewe additivity, was observed at the two lowest concentrations tested (D = 0.039) but not at higher concentrations due to a plateau in CCL2 expression, while synergistic CCL3 induction was observed at all concentrations (D = 0.623). To evaluate the effect of CMS in vivo, we next immunized mice with RBD-NP formulated with O/W or CMS:O/W (Fig. 9c). CMS enhanced both anti-RBD IgG1 and IgG2a Abs, with a more pronounced effect for IgG2a, a surrogate for Th1 immune responses in mice, than IgG1 (median fold: 42 vs 6.2, *p* < 0.001) (Fig. 9c). To further elucidate the mechanism of action of CMS, we stimulated the human monocytic cell line THP-1 stably expressing a secreted embryonic alkaline phosphatase reporter inducible by NF-κB with CMS. CMS induced NF-κB activation in THP-1 cells; however, this activation was not observed in THP-1 cells lacking MyD88 (Fig. 9d). Finally, we performed in vivo immunization with RBD-NP formulated with CMS:O/W in C57BL6 wild-type and *Myd88*^−/−^ mice (Fig. 9e). Importantly, impaired anti-RBD humoral response was demonstrated in *Myd88*^−/−^ mice in comparison to wild-type mice in the IgG2c subclass but not in IgG1 (Fig. 9e).

## Discussion

Despite rapid development and deployment of effective SARS-CoV-2 vaccines based on mRNA or viral vector technologies^1,2^, there remains an urgent global need for affordable, scalable, and practical coronavirus vaccines^3–6^. In this context, adjuvanted protein subunit vaccines may play an important role for increasing worldwide vaccine coverage, based in part on their generally robust history of safety and efficacy in special populations such as infants and older adults. Adjuvanted subunit vaccines consisting of Spike or RBD proteins, expressed in soluble or nanoparticle forms, are currently in different stages of pre-clinical and clinical development^7–13,15,31–36,39–41^. On-going efforts are required to define optimal combinations of antigens and adjuvants to enhance immunogenicity across the lifespan. Here, we assessed the immunogenicity of RBD-NP formulated with four different adjuvants, namely the MF59-like *AddaVax*, AS03-like *AddaS03*, AS01B, and CMS:O/W. Consistent with prior studies, we found that the first three elicited high levels of anti-RBD neutralized Abs^15,36,41^. However, CMS:O/W was the most effective at enhancing RBD-NP immunogenicity in both young and aged mice and protected the latter from live SARS-CoV-2 challenge. To provide a mechanistic basis for this phenomenon, we found that CMS:O/W promotes antigen retention in the dLN and induced gene expression profiles at both the injection site and the dLN that are distinct from the ones elicited by *AddaVax*, including induction of type I IFN-dependent ISGs in the dLN that may promote immunogenicity^69^. Further, we assessed the mechanistic role of each component of CMS:O/W in human PBMCs in vitro and in mice in vivo, demonstrating that CMS contributes to Th1 polarization via MyD88-dependent proinflammatory cytokine production. Overall, our study provides mechanistic and translational information on O/W adjuvants and might inform the development of adjuvanted RBD-NP vaccines effective across multiple age groups.

In this study, we compared the immunogenicity of monomeric RBD, Spike, and RBD-NP using the same adjuvant formulation, namely *AddaVax*. RBD-NP elicited higher Ab titers than soluble RBD monomer and was more immunogenic than soluble Spike trimer across all tested doses despite containing fewer epitopes. These differences translated into improved serum neutralizing activity upon immunization with *AddaVax*-adjuvanted RBD-NP as assessed by surrogate and live virus neutralization assay. We also compared in vivo efficacy of high-dose soluble RBD monomer (10 µg) and low-dose RBD-NP (0.3 µg) formulated with a same adjuvant and showed that aged mice immunized with CMS:O/W-adjuvanted RBD-NP are significantly more protected from SARS-CoV-2 challenge than mice immunized with CMS:O/W-adjuvanted soluble RBD with respect to weight loss, lung viral titer and lung inflammation. Our results demonstrating enhanced immunogenicity and protection of RBD-NP in comparison to RBD are consistent with the concept that antigen display onto NP scaffolds enhances immunogenicity and activates B cells to produce antigen-specific Abs^24–30^. However, a recent study has reported that immunization of non-human primates with RBD-NP or Spike Hexapro formulated with AS03 elicited comparable SARS-CoV-2 neutralizing titers^36^. Whether the differences between these findings and our study are due to use of different reagents (distinct RBD-NP designs, Spike vs Spike Hexapro, *AddaVax* vs AS03), doses and/or animal models (mice vs non-human primates) has not yet been determined. Nevertheless, both studies as well as additional recent publications support the use of RBD-NP as an effective SARS-CoV-2 vaccine antigen^13,15,31–34,36,40,41^. Although we focused on neutralizing Ab as an important correlate of protection against SARS-CoV-2 infection^57–59^, further studies are needed to assess the formation and longevity of germinal centers which are important for the induction of a high quality and long lasting protective immune response.

While generation of RBD-NP by fusing RBD directly to LuS resulted in poor yields, we successfully designed a two-component NP that can assemble via a SpyTag/SpyCatcher technology. As multiple two component NPs have demonstrated efficacy in preclinical and clinical studies^15,31,34,36,47,70,71^, we pursued this system that efficiently yielded pure RBD-NP. We confirmed the proper display of RBD onto NPs via ELISA employing hACE2 and anti-RBD mAbs. Interestingly, we observed preferential binding of mAbs to RBD-NP under low coating concentrations of RBD, Spike, and RBD-NP. Increased Ab binding at low antigen concentrations may be explained by increased avidity due to high-density RBD display on the NP core. However, as these proteins (i.e., RBD, Spike, and RBD-NP) contained non-equimolar concentrations of RBD, and binding capacity of each protein to ELISA plates might differ, further studies will be needed for precise assessment of Ab avidity relative to RBD display. Although our study did not fully characterize RBD-NP (e.g., loading efficiency, RBD:LuS ratio, and protein stability), it did establish a potent research tool enabling proof-of-concept studies to identify and characterize optimal combinations of adjuvants and antigens leveraging already characterized antigen designs (i.e., RBD monomer, Spike trimer, SpyCatcher/SpyTag system to generate RBD-NP). Overall, our work demonstrated the efficacy and mechanistic characterization of a CMS:O/W-adjuvanted RBD-NP. Nevertheless, additional protein characterization studies are needed to better characterize RBD-NPs to enable further translation and eventual clinical trials.

As expected, immunization with the RBD-NP alone did not induce robust immune response and required adjuvants to confer significant protection. Our observations are consistent with results of a human clinical trial that evaluated a self-assembling, two-component RBD-NP with non-adjuvanted or oil-in-water adjuvant AS03 formulation which demonstrated limited immunogenicity in the non-adjuvanted RBD-NP group^42^. Our study thus highlights the importance of adjuvantation as an approach to enable robust immunogenicity of NPs and the value of directly comparing multiple adjuvants to identify optimal adjuvant formulations to enhance immunity towards a vaccinal antigen in key vulnerable populations such as the older adults. To further optimize RBD-NP immunogenicity, we compared four adjuvant formulations, namely *AddaVax*, *AddaS03*, AS01B, and CMS:O/W. The first three (or similar adjuvant formulations) increase RBD-NP immunogenicity^15,36,41^, with an AS03-adjuvanted RBD-NP being currently evaluated in a clinical trial (NCT04750343). CMS:O/W is a potent adjuvant with a low reactogenicity profile^52^ but until now had never been tested with an RBD-NP. The CMS:O/W adjuvant technology is a third generation of the so-called carbohydrate fatty acid sulfate esters-based adjuvants such as CoVaccine HT^72^. Compared to CoVaccine HT, CMS:O/W has an improved safety profile but similar ability to induce robust Ab titers. Strikingly, CMS:O/W induced the highest levels of anti-RBD IgG Abs in young and aged mice by enhancing both anti-RBD IgG1 and IgG2a with a favorable reactogenicity profile. This translated into high SARS-CoV-2 neutralizing titers that protected aged mice from SARS-CoV-2 challenge. Serum samples of mice immunized with RBD-NP formulated with CMS:O/W also demonstrated the highest neutralization titers against B.1.1.7 and B.1.351 pseudoviruses which represent SARS-CoV-2 variants of concern. Although CMS:O/W is a promising adjuvant formulation for an RBD-NP-based SARS-CoV-2 vaccine effective across multiple age groups, further studies comparing to emulsion-based adjuvants such as GLA-SE or AS02 would be valuable to better characterize the relative performance of the CMS:O/W adjuvant.

To define a mechanistic basis for CMS adjuvanticity, we hypothesized that CMS:O/W could induce significant local cytokine and chemokine production at the injection site and/or antigen retention in the dLN. We focused on these mechanisms as they have been described previously for O/W emulsions^66,67^. By comparing CMS:O/W and *AddaVax* adjuvants, we found that both induced high antigen retention in the dLN and expression of pro-inflammatory genes at the injection site. Although we demonstrated that these O/W emulsions induced high antigen retention in the dLN by using R-PE as a model antigen, the antigen retention properties of the adjuvants may be less significant with RBD-NP due to its larger size. Future studies should further characterize the antigen retention effect of CMS:O/W in the dLN. Cytokine and chemokine production at the injection site can promote innate immune cell recruitment, activation, and subsequent antigen presentation^66^. However, only CMS:O/W enhanced type I IFN-dependent ISG expression in the dLN which can promote differentiation of CD4^+^ T follicular helper cells and therefore antigen-specific Ab response^69^. By studying each component of CMS:O/W individually, we found that CMS contributes to a favorable Th1 immune response in a MyD88-dependent manner. Synergistic induction of the chemokine CCL2 and CCL3 that attracts monocytes, DCs and memory T cells, as well as polymorphonuclear leukocytes^73^, respectively to sites of infection/immunization may contribute to the adjuvanticity of CMS:O/W. Further work will be required to define the precise mechanism of action of CMS:O/W.

The emergence of SARS-CoV-2 variants that can escape neutralizing Abs elicited by infection or vaccination has raised concerns regarding possible reduction in vaccine efficacy^62–64^. While our results show significant neutralization of B.1.1.7 and B.1.351 pseudoviruses by serum samples of mice immunized with RBD-NP and CMS:O/W, there is need for rapid development of vaccines specifically targeting SARS-CoV-2 variants of concern^74^. In addition, the risk posed by emerging zoonotic coronaviruses calls for the development of pan-coronavirus vaccines that can protect against circulating variants as well as strains currently circulating only in non-human animals but that can generate future human outbreaks and pandemics. The SpyTag/SpyCatcher conjugation platform employed in our study has recently been used to generate mosaic NP displaying RBD proteins derived from up to 8 strains^31^. Whether different adjuvant formulations (e.g., *AddaVax*, CMS:O/W) can modulate the breadth of the Ab response elicited by mosaic RBD-NP is an important area for future research.

Overall, we have identified a CMS:O/W-adjuvanted RBD-NP vaccine formulation that potently induces cross-neutralizing Abs and protective immunity across multiple age groups and have provided a mechanistic basis for its enhanced immunogenicity. Our approach may inform further pre-clinical and clinical development of precision adjuvanted RBD-NP-based SARS-CoV-2 and pan-coronavirus vaccines tailored for robust immunogenicity and protection in vulnerable older adults.

## Methods

### Protein expression and purification

Full-length SARS-CoV-2 spike glycoprotein (M1-Q1208, GenBank MN90894) and RBD constructs (amino acid residues R319-K529, GenBank MN975262.1), both with an HRV3C protease cleavage site, a TwinStrepTag and an 8XHisTag at C-terminus, were obtained from Barney S. Graham (NIH Vaccine Research Center) and Aaron Schmidt (Ragon Institute), respectively. To generate RBD-Catch and LuS-Tag constructs in a mammalian expression vector, SARS-CoV-2 RBD and SpyCatcher^53^ were fused by a GGSGGS linker for RBD-Catch, and N-terminal Spy-tag was added to lumazine synthase from *Aquifex aeolicus* bearing D71N mutation for LuS-Tag. Both constructs contain a signal peptide (MKHLWFFLLLVAAPRWVLS) at N-terminus and HRV3C protease site, followed by a TwinStrepTag at C-terminus. These mammalian expression vectors were used to transfect Expi293F suspension cells (Thermo Fisher) using polyethylenimine (Polysciences). Cells were allowed to grow at 37 °C, 8% CO_2_ for additional 5 days before harvesting for purification. Protein was purified in a PBS buffer (pH 7.4) from filtered supernatants by using either StrepTactin resin (IBA) or Cobalt-TALON resin (Takara). Affinity tags were cleaved off from eluted protein samples by HRV 3C protease, and tag-removed proteins were further purified by size-exclusion chromatography using a Superose 6 10/300 column (Cytiva) for full-length Spike and a Superdex 200 10/300 Increase 10/300 GL column (Cytiva) for RBD, RBD-Catch, and LuS-Tag in a PBS buffer (pH 7.4).

### RBD-Catch and LuS‐Tag conjugations

To saturate Lus-Tag surface with RBD, a 1:1.2 molar ratio of LuS-Tag and RBD-Catch components were mixed at 40 µM of LuS-Tag in a PBS buffer (pH 7.4) and incubated at room temperature for approximately 1 h. Reaction mixture was applied to a Superdex200 Increase 10/300 GL column (Cytiva) in a PBS buffer (pH 7.4) to purify RBD-nanoparticles from unconjugated RBD-Catch. The conjugated RBD-nanoparticle product was confirmed by SDS-PAGE and analyzed by negative-stain EM.

### SDS-PAGE analysis

Proteins samples (250 µg/ml) in NuPAGE LDS Sample Buffer (Invitrogen) were heated to 95 °C for 5 min, and 10 µl (2.5 µg) were loaded to a NuPAGE 10% Bis-Tris gel (Invitrogen). The gel was run in NuPAGE MOPS Buffer (Invitrogen) at 60 V for 45 min and then 110 V for 105 min. The gel was then sequentially rinsed as follows prior to imaging: (1) with DI water and fixed for 15 min in 50 mL of 40% ethanol and 10% acetic acid fixing solution; (2) with DI water and incubated with QC Colloidal Coomassie Blue (Bio-Rad) on a rotating shaker for 1 h at RT; and (3) twice with DI water and incubated on a rotating shaker for 75 min, changing the water every 15 min. Employing PNGase F kit (New England BioLabs), 3.5 µg of protein samples were diluted with 1 µl Glycoprotein Denaturing Buffer and 5.5 µl DI water to a total volume of 10 µl. Samples were then heated to 100 °C for 10 min and chilled on ice. 2 µl of GlycoBuffer 2, 2 µl of NP-40, and 6 µl of DI water were added, followed by 1 µl of PNGase F. In non-PNGase F controls, DI water was added in place of PNGase F. Samples were incubated at 37 °C for 2 h and then prepared as before with LDS Sample Buffer, at a final concentration of 125 µg/ml. Gels were run as before but with 1.25 µg of protein loaded per well.

### Negative staining electron microscopy

Purified RBD-nanoparticle samples were diluted to 0.01–0.05 mg/mL with a PBS (pH 7.4) buffer. A 4-µl drop of the diluted sample was applied to a freshly glow-discharged carbon-coated copper grid (400-mesh, EMS) for approximately 1 min. The drop was removed using blotting paper, and the grid was washed three times with 5-µl drops of the same buffer. Adsorbed proteins were negatively stained by soaking in 4-µl drops of 2% uranyl acetate for approximately 10 s and removing drop with filter paper. Micrographs were collected using JEM-1400 Plus electron microscope (JEOL, USA) operated at 80 kV, resulting in ~0.15 nm/pixel at 80,000x magnification.

### Dynamic light scattering

Purified protein samples (250 μg/ml) were loaded into a disposable microcuvette and measured at 25 °C using a Zetasizer Ultra instrument (Malvern Panalytical) equipped with a 633-nm laser with 3 scans of 60 s each. Each sample was measured in triplicate, and the intensity of the size distribution was plotted in GraphPad Prism 9 (GraphPad Software).

### Enzyme-Linked Immunosorbent Assay (ELISA)

ELISA was employed to examine the binding ability of the purified proteins to hACE2 and RBD-specific monoclonal Abs (mAbs). RBD monomer, Spike trimer, RBD-Catch, LuS-Tag, and RBD-NPs were respectively diluted to concentrations of 0.5 and 5 μg/ml for mAb binding and 1 µg/ml for hACE2 binding, and 50 μl/well were added to coat 96-well high-binding flat-bottom plate (Corning) overnight at 4 °C. Plates were washed with 0.05% Tween 20 PBS (PBS-T) and blocked with 1% BSA PBS for 1 h at room temperature (RT). The plates were then incubated with sequentially 1:5 serially diluted hACE2-Fc (InvivoGen) and two anti-SARS-CoV-2 RBD mAbs (clones H4 [InvivoGen] and CR3022 [Abcam]) starting at 10 μg/ml in blocking buffer. After 2 h of incubation, the plates were washed with PBS-T three times and incubated with HRP-conjugated detection Abs (mouse anti-human IgG1 Fc-HRP, Southern Biotec). Plates were washed five times and developed with tetramethylbenzidine (OptEIA Substrate Solution, BD Biosciences) for 5 min, then stopped with 2 N H_2_SO_4_. Optical densities (ODs) were read at 450 nm with a SpectraMax iD3 microplate reader (Molecular Devices).

### RBD-NP stability analysis

RBD-NP samples (1 mg/ml) were subjected to one to five cycles of freeze-thaw cycles by storing in a −80 °C freezer for at least 1 day, followed by incubation at RT for 30 min. For the storage temperature study, RBD-NP samples (1 mg/ml) were incubated at 4 °C or RT for 5–7 days. The RBD-NP samples were then analyzed by ELISA.

### Live SARS-CoV-2 in vitro competition assay

The day prior to infection, 5 × 10^3^ VeroE6 cells were plated per well in DMEM (Quality Biological) supplemented with 10% v/v fetal bovine serum (Gibco), 1% v/v Penicillin-Streptomycin (Gemini-Bio), and 1% v/v L-Glutamine (Gibco). 1 mg/ml stock concentrations of SARS-CoV-2 Spike, SARS-CoV-2 RBD, and SARS-CoV-2 RBD-NP were diluted to 50 μg/ml in 400 μl complete VeroE6 media in a 96-well dilution block in duplicate and then serially diluted down the plate 1:3 to produce an 8-point dilution curve (125 μl into 250 μl media). Media was removed from the VeroE6 cells, and 90 μl of each dilution was then transferred to the cells and left to incubate at 37 °C and 5% CO_2_ for 2 h. After incubation, each well was infected with a 0.1 M.O.I. of SARS-CoV-2 ΔORF7a::GFP (provided by Dr. Ralph Baric (UNC)) diluted in 10 μl media. A parallel plate was left uninfected to monitor cytotoxicity. After 48 h, the infected plates were fixed in 4% paraformaldehyde for 1 h, Hoechst-stained, and read on a plate reader (Nexcelom Biosciences, Lawrence, MA). The percentage of GFP+ cells in each well was counted and compared to an untreated, infected control to give an inhibitory concentration of 50 (IC_50_) for each protein. The parallel cytotoxicity plate was analyzed with Cell Titer Glo (Promega, Madison, WI) and read on a BioTek Synergy HTX plate reader (BioTek Instruments, Inc., Winooski, VT). Cell viability was compared to the untreated control.

### Animals

Female, 3-month-old BALB/c and C57BL/6 J mice were purchased from Jackson Laboratory (Bar Harbor, ME), and CD-1 mice were purchased from Charles River Laboratories (Wilmington, MA). Female, 11–13 months old BALB/c mice purchased from Taconic Biosciences (Germantown, NY) were used for aged mice experiments. Female, 6–8 weeks old wild-type (#000664) and *Myd88*^−*/*−^ (#009088) C57BL/6 were mice purchased from The Jackson Laboratory (Bar Harbor, ME). Mice were housed under specific pathogen-free conditions at Boston Children’s Hospital, and all procedures were approved under the Institutional Animal Care and Use Committee (IACUC) and operated under the supervision of the Department of Animal Resources at Children’s Hospital (ARCH) (Protocol number 19-02-3897R). At the University of Maryland School of Medicine, mice were housed in a biosafety level 3 (BSL3) facility for all SARS-CoV-2 infections with all procedures approved under the IACUC (Protocol number #1120004).

### Mouse immunization

All formulations for immunization were prepared under sterile conditions. Mice were injected with antigens (RBD monomer, Spike trimer, and RBD-NPs), with or without adjuvants. Mock treatment mice received phosphate-buffered saline (PBS) alone. Injections (50 µl) were administered intramuscularly in the caudal thigh on Days 0 and 14. The SARS-CoV-2 antigens and their doses employed were: RBD monomer (0.3, 1, and 3 µg in Figs. 2–3, and 10 µg in Fig. 5), Spike trimer (0.3, 1, and 3 µg in Figs. 2–3), and RBD-NPs (0.3, 1, and 3 µg in Figs. 2–3, and 0.3 µg in Figs. 4–7). The adjuvants studied and their doses employed were: *AddaVax* (25 µl), *AddaS03* (25 µl) (InvivoGen), AS01B (40 µl) (obtained from the *Shingrix* vaccine, GSK Biologicals SA, Belgium), and CMS:O/W adjuvant (25 µl corresponding with 1 mg of CMS) (LiteVax, The Netherlands).

### Mouse serum antibody ELISA

RBD- and Spike-specific Ab titers were quantified in serum samples by ELISA. High-binding flat-bottom 96-well plates (Corning) were coated with 50 ng/well RBD or 25 ng/well Spike and incubated overnight at 4 °C. Plates were washed with PBS-T (PBS + 0.05% Tween 20) and blocked with 1% BSA PBS for 1 h at RT. Serum samples were serially diluted 4-fold from 1:100 up to 1:1.05^8^ and then incubated for 2 h at RT. Plates were washed three times and incubated for 1 h at RT with HRP-conjugated anti-mouse IgG, IgG1, IgG2a, or IgG2c (Southern Biotech). Plates were washed five times and developed with tetramethylbenzidine (1-Step Ultra TMB-ELISA Substrate Solution, ThermoFisher, for RBD-ELISA, and BD OptEIA Substrate Solution, BD Biosciences, for Spike ELISA) for 5 min, then stopped with 2 N H_2_SO_4_. Optical densities (ODs) were read at 450 nm with a SpectraMax iD3 microplate reader (Molecular Devices). End-point titers were calculated as the dilution that emitted an optical density exceeding a 3× background. An arbitrary value of 25 was assigned to samples with OD values below the limit of detection for which it was not possible to interpolate the titer.

### Surrogate of virus neutralization test (sVNT)

We performed sVNT to measure the degree of hACE2/RBD inhibition by immune sera. High-binding flat-bottom 96-well plates (Corning, NY) were coated with 100 ng/well recombinant human ACE2 (hACE2) (Sigma-Aldrich) in PBS, incubated overnight at 4 °C, washed three times with PBS-T, and blocked with 1% BSA PBS for 1 h at RT. Each serum sample was diluted at 1:160, pre-incubated with 3 ng of RBD-Fc in 1% BSA PBS for 1 h at RT, and then transferred to the hACE2-coated plate. RBD-Fc without pre-incubation with serum samples was added as a positive control, and 1% BSA PBS without serum pre-incubation was added as a negative control. Plates were then washed three times and incubated with HRP-conjugated anti-human IgG Fc (Southern Biotech) for 1 h at RT. Plates were washed five times and developed with tetramethylbenzidine (BD OptEIA Substrate Solution, BD Biosciences) for 5 min, then stopped with 2 N H_2_SO_4_. The optical density was read at 450 nm with a SpectraMax iD3 microplate reader (Molecular Devices). Percentage inhibition of RBD binding to hACE2 was calculated with the following formula: Inhibition (%) = [1 – (Sample OD value – Negative Control OD value)/(Positive Control OD value – Negative Control OD value)] × 100.

### Live SARS-CoV-2 virus neutralization test

All serum samples were heat-inactivated at 56 °C for 30 min to deactivate complement and allowed to equilibrate to RT prior to processing for neutralization titer. Samples were diluted in duplicate to an initial dilution of 1:20 followed by 1:2 serial dilutions (vaccinated samples), resulting in a 12-dilution series with each well containing 60 µl. All dilutions employed DMEM (Quality Biological), supplemented with 10% (v/v) fetal bovine serum (heat-inactivated, Gibco), 1% (v/v) penicillin/streptomycin (Gemini Bio-products), and 1% (v/v) L-glutamine (2 mM final concentration, Gibco). Dilution plates were then transported into the BSL-3 laboratory, and 60 µl of diluted SARS-CoV-2 (WA-1, courtesy of Dr. Natalie Thornburg/CDC) inoculum was added to each well to result in a multiplicity of infection (MOI) of 0.01 upon transfer to titering plates. A non-treated, virus-only control and mock infection control were included on every plate. The sample/virus mixture was then incubated at 37 °C (5.0% CO_2_) for 1 h before transferring 100 µl to 96-well titer plates with 5e3 VeroE6 cells. Titer plates were incubated at 37 °C (5.0% CO_2_) for 72 h, followed by cytopathic effect (CPE) determination for each well in the plate. The first sample dilution to show CPE was reported as the minimum sample dilution required to neutralize >99% of the concentration of SARS-CoV-2 tested (NT99).

### Pseudovirusneutralization test

The SARS-CoV-2 pseudoviruses expressing a luciferase reporter gene were generated as follows. The packaging plasmid psPAX2 (AIDS Resource and Reagent Program), luciferase reporter plasmid pLenti-CMV Puro-Luc (Addgene), and spike protein expressing pcDNA3.1-SARS CoV-2 SΔCT of variants were co-transfected into HEK293T cells by lipofectamine 2000 (ThermoFisher). Pseudoviruses of SARS-CoV-2 variants were generated by using WA1/2020 strain (Wuhan/WIV04/2019, GISAID accession ID: EPI_ISL_402124), B.1.1.7 variant (GISAID accession ID: EPI_ISL_601443), or B.1.351 variant (GISAID accession ID: EPI_ISL_712096). The supernatants containing the pseudotype viruses were collected 48 h post-transfection and were purified by centrifugation and filtration with 0.45 µm filter. To determine the neutralization activity of the plasma or serum samples from participants, HEK293T-hACE2 cells were seeded in 96-well tissue culture plates at a density of 1.75 × 10^4^ cells/well overnight. Three-fold serial dilutions of heat-inactivated serum or plasma samples were prepared and mixed with 50 µL of pseudovirus. The mixture was incubated at 37 °C for 1 h before adding to HEK293T-hACE2 cells. 48 h after infection, cells were lysed in Steady-Glo Luciferase Assay (Promega). SARS-CoV-2 neutralization titers were defined as the sample dilution at which a 50% reduction in relative light units (RLU) was observed relative to the average of the virus control wells.

### Splenocyte restimulation, intracellular cytokine staining, and flow cytometry

Mouse spleens were mechanically dissociated and filtered through a 70 µm cell strainer. After centrifugation, cells were treated with 1 mL ammonium-chloride-potassium lysis buffer for 2 min at RT. Cells were washed and plated in a 96-well U-bottom plate (2 × 10^6^/well) and incubated overnight in RPMI 1640 supplemented with 10% heat-inactivated FBS, penicillin (100 U/ml), streptomycin (100 mg/ml), 2-mercaptoethanol (55 mM), non-essential amino acids (60 mM), HEPES (11 mM), and L-Glutamine (800 mM) (all Gibco). Next day, SARS-CoV-2 spike RBD peptide pools (PM-WCPV-S-RBD-1, JPT) were added at 0.6 nmol/ml in the presence of anti-mouse CD28 and CD49d (1 μg/mL, BD, #553295 and #553313) and brefeldin A (5 μg/ml, BioLegend, #420601). PMA and ionomycin (BioLegend, #423301) were used as positive controls. After 6 h stimulation, cells were washed twice and were treated with Mouse Fc Block (BD). Cells were washed and stained with Aqua Live/Dead stain (Life Technologies, 1:500) for 15 min at RT. Following two additional washes, cells were incubated with the following Abs for 30 min at 4 °C: anti-mouse CD44 [IM7, PerCP-Cy5.5, BioLegend #103032, 1:160], anti-mouse CD3 [17A2, Brilliant Violet 785, BioLegend #100232, 1:40], anti-mouse CD4 [RM4-5, APC/Fire 750, BioLegend #100568, 1:160] and anti-mouse CD8 [53–6.7, Brilliant UltraViolet 395, BD #563786, 1:80]. After washing with PBS, cells were fixed and permeabilized by using the BD Cytofix/Cytoperm kit and were subjected to intracellular staining (30 min at 4 °C) using the following Abs: anti-mouse IFNγ [XMG1.2, Alexa Fluor 488, BioLegend #505813, 1:160], anti-mouse TNF [MP6-XT22, PE Cy7, BioLegend #506324, 1:160], anti-mouse IL-2 [JES6–5H4, PE, BioLegend #503808, 1:40], anti-mouse IL-4 [11B11, APC, BioLegend #504106, 1:40] and anti-mouse IL-5 [TRFK5, APC, BioLegend #504306, 1:40]. Finally, cells were fixed in 1% paraformaldehyde (Electron Microscopy Sciences) for 20 min at 4 °C and stored in PBS at 4 °C until acquisition. Samples were analyzed on an LSR II (BD) flow cytometer and FlowJo v10.8.1 (FlowJo LLC).

### SARS-CoV-2 mouse challenge study

Mice were anesthetized by intraperitoneal injection of 50 μL of a xylazine and ketamine mix (0.38 mg/mouse and 1.3 mg/mouse, respectively) diluted in PBS. Mice were then inoculated intranasally with 1 × 10^3^ PFU of mouse-adapted SARS-CoV-2 (MA10, courtesy of Dr. Ralph Baric (UNC)) in 50 μl divided between nares^60^. Challenged mice were weighed on the day of infection and daily for up to 4 days post-infection. At 4-days post-infection, mice were sacrificed, and lungs were collected to assess virus load by plaque assay and gene expression profiles. SARS-CoV-2 lung titers were quantified by homogenizing harvested lungs in PBS (Quality Biological Inc.) using 1.0 mm glass beads (Sigma Aldrich) and a Beadruptor (Omni International Inc.). Homogenates were added to Vero E6 cells and SARS-CoV-2 virus titers were determined by counting plaque-forming units (pfu) using a 6-point dilution curve. RNA was isolated from lung homogenates using a Direct-zol RNA miniprep kit (Zymo Research). RNA concentration and purity (260/280 and 260/230 ratios) were measured by NanoDrop (ThermoFisher Scientific). For histopathology analysis, slides were prepared as 5 μm sections and stained with hematoxylin and eosin.

### Analysis of inflammatory responses at injection site, dLN, and serum

Young (3-month-old) BALB/c mice were injected with PBS, *AddaVax* or CMS:O/W adjuvant, and their local muscle tissue, dLN, and serum samples were harvested for subsequent analysis 24 h later. For dLN analysis, adjuvants were injected in caudal thigh, and inguinal LNs were collected. For muscle tissue analysis, adjuvants were injected in the gastrocnemius muscle, and the whole gastrocnemius was collected. Samples were stored in RNA*later* (Invitrogen) for 24 h at 4 °C and then homogenized in TRI Reagent (Zymo Research) with a beadbeater. Samples were then centrifuged, and the clear supernatant was transferred to a new tube for subsequent RNA isolation. RNA was isolated from TRI Reagent samples using phenol-chloroform extraction or column-based extraction systems (Direct-zol RNA Miniprep, Zymo Research). RNA concentration and purity (260/280 and 260/230 ratios) were measured by NanoDrop (ThermoFisher Scientific). Cytokine and chemokine concentrations in serum samples were measured using customized Milliplex mouse magnetic bead panels (Milliplex). Assays were analyzed on the Luminex FLEXMAP 3D employing xPONENT software (Luminex) and Millipore Milliplex Analyst. Data were excluded from analysis if <30 beads were recovered.

### Gene expression analysis by qPCR

RNA was isolated from TRI Reagent samples using phenol-chloroform extraction or column-based extraction systems (Direct-zol RNA Miniprep, Zymo Research). RNA concentration and purity (260/280 and 260/230 ratios) were measured by NanoDrop (Thermo Fisher Scientific). Samples with an A260/A280 ratio of <1.7 were excluded for further analysis. For lymph nodes and muscles, purified RNA was analyzed for gene expression by qPCR on a CFX384 real-time cycler (Bio-rad) using pre-designed KiCqStart SYBR Green Primers (MilliporeSigma) specific for *Csf2* (RM1_Csf2 and FM1_Csf2), *Cxcl9* (RM1_Cxcl9 and FM1_Cxcl9), *Ifit2* (RM1_Ifit2 and FM1_Ifit2), *Rsad2* (RM1_Rsad2 and FM1_Rsad2), *Il6* (RM1_Il6 and FM1_Il6), *Cxcl1* (RM1_Cxcl1 and FM1_Cxcl1), *Rpl13a* (RM1_Rpl13a and FM1_Rpl13a). For lung tissues, cDNA was prepared from purified RNA with RT^2^ First Strand Kit (Qiagen). cDNA was quantified by qPCR on a 7300 real-time PCR system (Applied Biosystems – Life Technologies) using pre-designed SYBR Green Primers (QIAGEN) specific for *Ifit2* (PPM05993A), *Rsad2* (PPM26539A), *Il6* (PPM03015A), and *Rpl13a* (PPM03694A).

### Measurement of antigen retention within the dLN

Young (3-month-old) BALB/c mice were injected IM with vaccine formulation (50 µL) applying R-phycoerythrin (R-PE) as a model antigen (6 µg). 24 h later, the dLN was collected and homogenized in water with a beadbeater. Fluorescence values were measured with SpectraMax i3x microplate reader (Molecular Devices) and expressed as arbitrary units after background (deionized water) subtraction.

### Human PBMC isolation

Human peripheral blood was collected from healthy adult study participants 18–40 years of age per a Boston Children’s IRB-approved protocol (protocol number X07-05-0223). All participants signed an informed consent form prior to enrollment. Heparinized whole blood was centrifuged at 500 g for 10 min, prior to removal of the upper layer of platelet-rich plasma. Plasma was centrifuged at 3000 g for 10 min, and platelet-poor plasma (PPP) was collected and stored on ice. The remaining blood was reconstituted to its original volume with heparinized Dulbecco’s PBS and layered on Ficoll-Paque gradients (Cytiva) in Accuspin tubes (Sigma-Aldrich). PBMCs were collected after centrifugation and washed twice with PBS.

### Human PBMC stimulation

PBMCs were resuspended at a concentration of 2 × 10^5^ cells per well in a 96-well U-bottom plate (Corning) in 200 µL RPMI-1640 media (Gibco) supplemented with 10% autologous PPP, 100 IU/mL penicillin, 100 µg/mL streptomycin, and 2 mM L-glutamine. PBMCs were incubated for 24 h at 37 °C in a humidified incubator at 5% CO_2_ with indicated treatments. After culture, plates were centrifuged at 500 g and supernatants were removed by pipetting without disturbing the cell pellet. Cytokine production was measured in cell culture supernatants using customized Milliplex human cytokine magnetic bead panels (Milliplex). Assays were analyzed on the Luminex FLEXMAP 3D employing xPONENT software (Luminex) and Millipore Milliplex Analyst. Cytokine measurements were excluded from analysis if <30 beads were recovered. CCL2 and CCL3 were measured by ELISA kits (Invitrogen).

### THP1 cell stimulation

THP1-Dual and THP1-Dual KO-MyD reporter cells (Invivogen) were resuspended at a concentration of 100,000 cells per well in a 96-well U-bottom plate (Corning) in 200 µl RPMI-1640 media (Gibco), supplemented with 10% fetal bovine serum (Gibco), 100 IU/ml penicillin, 100 µg/ml streptomycin, 2 mM L-glutamine, and 100 µg/ml Normocin (Invivogen). Cells were incubated for 20 h at 37 °C in a humidified incubator at 5% CO_2_ with indicated treatments. After culture, plates were centrifuged at 500 g, and supernatants were removed by pipetting without disturbing the cell pellet. To assess NF-κB activity via the conjugated SEAP reporter, 20 µl of supernatant were combined with 180 µl per well of QUANTI-Blue (Invivogen) in a clear 96-well flat-bottom plate (Corning) and incubated for 3.5 to 4 h at 37 °C. Optical density was read at 630 nm with a SpectraMax iD3 microplate reader (Molecular Devices).

### Statistical analysis

Statistical analyses employed Prism v9.0.2 (GraphPad Software). Some datasets were analyzed after Log-transformation as indicated in the figure legends. Statistical differences between groups in datasets with one categorical variable were evaluated by two sample *t* test (2 groups) or one-way ANOVA (more than 2 groups) corrected for multiple comparisons. Two-way ANOVA corrected for multiple comparisons and evaluated statistical differences between groups in datasets with two categorical variables. *p* < 0.05 were considered significant.

### Reporting summary

Further information on research design is available in the Nature Portfolio Reporting Summary linked to this article.

## Supplementary information

Supplementary Information

Supplementary Data

REPORTING SUMMARY

## Data Availability

The source data underlying Figs. 1c–e, 2–4, 5a–e, 6–9 are provided as Supplementary Data.

## References

[1] Graham BS (2020). Rapid COVID-19 vaccine development. Science.

[2] Gebre MS (2021). Novel approaches for vaccine development. Cell.

[3] Koff WC (2021). Development and deployment of COVID-19 vaccines for those most vulnerable. Sci. Transl. Med.

[4] Katz IT, Weintraub R, Bekker LG, Brandt AM (2021). From vaccine nationalism to vaccine equity—finding a path forward. N. Engl. J. Med.

[5] Mejia, R., Hotez, P. & Bottazzi, M. E. Global COVID-19 efforts as the platform to achieving the sustainable development goals. *Curr. Trop. Med. Rep*. 1–5 (2020). 10.1007/s40475-020-00209-y 10.1007/s40475-020-00209-y PMC7440684 32844081

[6] Lancet Commission on, C.-V. & Therapeutics Task Force, M. Urgent needs of low-income and middle-income countries for COVID-19 vaccines and therapeutics. *Lancet* **397**, 562–564 (2021). 10.1016/S0140-6736(21)00242-7 PMC7906712 33516284

[7] Keech C (2020). Phase 1-2 trial of a SARS-CoV-2 recombinant spike protein nanoparticle vaccine. N. Engl. J. Med.

[8] Richmond P (2021). Safety and immunogenicity of S-Trimer (SCB-2019), a protein subunit vaccine candidate for COVID-19 in healthy adults: a phase 1, randomised, double-blind, placebo-controlled trial. Lancet.

[9] Tian JH (2021). SARS-CoV-2 spike glycoprotein vaccine candidate NVX-CoV2373 immunogenicity in baboons and protection in mice. Nat. Commun..

[10] Pollet, J. et al. SARSCoV-2 RBD219-N1C1: A yeast-expressed SARS-CoV-2 recombinant receptor-binding domain candidate vaccine stimulates virus neutralizing antibodies and T-cell immunity in mice. *Hum. Vaccin. Immunother*. 1–11 (2021). 10.1080/21645515.2021.1901545 10.1080/21645515.2021.1901545 PMC8054496 33847226

[11] Sridhar S (2022). Safety and immunogenicity of an AS03-adjuvanted SARS-CoV-2 recombinant protein vaccine (CoV2 preS dTM) in healthy adults: interim findings from a phase 2, randomised, dose-finding, multicentre study. Lancet Infect. Dis..

[12] Heath PT (2021). Safety and efficacy of NVX-CoV2373 Covid-19 vaccine. N. Engl. J. Med.

[13] Dalvie NC (2021). Engineered SARS-CoV-2 receptor binding domain improves manufacturability in yeast and immunogenicity in mice. Proc. Natl Acad. Sci. USA.

[14] Nanishi E (2022). An aluminum hydroxide:CpG adjuvant enhances protection elicited by a SARS-CoV-2 receptor binding domain vaccine in aged mice. Sci. Transl. Med..

[15] Walls AC (2020). Elicitation of potent neutralizing antibody responses by designed protein nanoparticle vaccines for SARS-CoV-2. Cell.

[16] Walls AC (2020). Structure, function, and antigenicity of the SARS-CoV-2 spike glycoprotein. Cell.

[17] Lan J (2020). Structure of the SARS-CoV-2 spike receptor-binding domain bound to the ACE2 receptor. Nature.

[18] Yan R (2020). Structural basis for the recognition of SARS-CoV-2 by full-length human ACE2. Science.

[19] Chen WH (2021). Genetic modification to design a stable yeast-expressed recombinant SARS-CoV-2 receptor binding domain as a COVID-19 vaccine candidate. Biochim. Biophys. Acta Gen. Subj..

[20] Piccoli L (2020). Mapping neutralizing and immunodominant sites on the SARS-CoV-2 spike receptor-binding domain by structure-guided high-resolution serology. Cell.

[21] Premkumar L (2020). The receptor binding domain of the viral spike protein is an immunodominant and highly specific target of antibodies in SARS-CoV-2 patients. Sci. Immunol..

[22] Yang J (2020). A vaccine targeting the RBD of the S protein of SARS-CoV-2 induces protective immunity. Nature.

[23] Dalvie NC (2022). Scalable, methanol-free manufacturing of the SARS-CoV-2 receptor-binding domain in engineered Komagataella phaffii. Biotechnol. Bioeng..

[24] Graham BS, Gilman MSA, McLellan JS (2019). Structure-based vaccine antigen design. Annu Rev. Med.

[25] Singh A (2021). Eliciting B cell immunity against infectious diseases using nanovaccines. Nat. Nanotechnol..

[26] Kwong PD, DeKosky BJ, Ulmer JB (2020). Antibody-guided structure-based vaccines. Semin Immunol..

[27] Ward AB, Wilson IA (2020). Innovations in structure-based antigen design and immune monitoring for next generation vaccines. Curr. Opin. Immunol..

[28] Brune KD, Howarth M (2018). New routes and opportunities for modular construction of particulate vaccines: stick, click, and glue. Front Immunol..

[29] Lopez-Sagaseta J, Malito E, Rappuoli R, Bottomley MJ (2016). Self-assembling protein nanoparticles in the design of vaccines. Comput Struct. Biotechnol. J..

[30] Irvine DJ, Read BJ (2020). Shaping humoral immunity to vaccines through antigen-displaying nanoparticles. Curr. Opin. Immunol..

[31] Cohen AA (2021). Mosaic nanoparticles elicit cross-reactive immune responses to zoonotic coronaviruses in mice. Science.

[32] He L (2021). Single-component, self-assembling, protein nanoparticles presenting the receptor binding domain and stabilized spike as SARS-CoV-2 vaccine candidates. Sci. Adv..

[33] Ma X (2020). Nanoparticle vaccines based on the receptor binding domain (RBD) and heptad repeat (HR) of SARS-CoV-2 elicit robust protective immune responses. Immunity.

[34] Tan TK (2021). A COVID-19 vaccine candidate using SpyCatcher multimerization of the SARS-CoV-2 spike protein receptor-binding domain induces potent neutralising antibody responses. Nat. Commun..

[35] Dai L (2020). A universal design of betacoronavirus vaccines against COVID-19, MERS, and SARS. Cell.

[36] Arunachalam PS (2021). Adjuvanting a subunit COVID-19 vaccine to induce protective immunity. Nature.

[37] Sahin U (2020). COVID-19 vaccine BNT162b1 elicits human antibody and TH1 T cell responses. Nature.

[38] Walsh EE (2020). Safety and immunogenicity of two RNA-based Covid-19 vaccine candidates. N. Engl. J. Med.

[39] Hauser BM (2022). Rationally designed immunogens enable immune focusing following SARS-CoV-2 spike imprinting. Cell Rep..

[40] Saunders KO (2021). Neutralizing antibody vaccine for pandemic and pre-emergent coronaviruses. Nature.

[41] King, H. A. D. et al. Efficacy and breadth of adjuvanted SARS-CoV-2 receptor-binding domain nanoparticle vaccine in macaques. *Proc. Natl Acad. Sci. USA* **118** (2021). 10.1073/pnas.2106433118 10.1073/pnas.2106433118 PMC8463842 34470866

[42] Song JY (2022). Safety and immunogenicity of a SARS-CoV-2 recombinant protein nanoparticle vaccine (GBP510) adjuvanted with AS03: A randomised, placebo-controlled, observer-blinded phase 1/2 trial. EClinicalMedicine.

[43] O’Hagan DT, Lodaya RN, Lofano G (2020). The continued advance of vaccine adjuvants—‘we can work it out’. Semin Immunol..

[44] Nanishi E, Dowling DJ, Levy O (2020). Toward precision adjuvants: optimizing science and safety. Curr. Opin. Pediatr..

[45] Pulendran B, P SA, O’Hagan DT (2021). Emerging concepts in the science of vaccine adjuvants. Nat. Rev. Drug Disco..

[46] Irvine DJ, Aung A, Silva M (2020). Controlling timing and location in vaccines. Adv. Drug Deliv. Rev..

[47] GlaxoSmithKline. *SK bioscience and GSK’s adjuvanted COVID-19 vaccine candidate meets coprimary objectives in a phase III study; Biologics License Application submitted for SKYCovione™(GBP510/GSK adjuvant) in South Korea*, https://www.gsk.com/en-gb/media/press-releases/sk-bioscience-and-gsk-s-adjuvanted-covid-19-vaccine-candidate-meets-coprimary-objectives-in-a-phase-iii-study/ (2022).

[48] Nanishi E (2022). Precision vaccine adjuvants for older adults: a scoping review. Clin. Infect. Dis..

[49] Dowling DJ, Levy O (2022). A precision adjuvant approach to enhance SARS-CoV-2 vaccines optimized for immunologically distinct vulnerable populations. Clin. Infect. Dis..

[50] Zhang X, Meining W, Fischer M, Bacher A, Ladenstein R (2001). X-ray structure analysis and crystallographic refinement of lumazine synthase from the hyperthermophile Aquifex aeolicus at 1.6 A resolution: determinants of thermostability revealed from structural comparisons. J. Mol. Biol..

[51] Wrapp D (2020). Cryo-EM structure of the 2019-nCoV spike in the prefusion conformation. Science.

[52] Hilgers LAT (2017). Carbohydrate fatty acid monosulphate esters are safe and effective adjuvants for humoral responses. Vaccine.

[53] Brune KD (2016). Plug-and-Display: decoration of Virus-Like Particles via isopeptide bonds for modular immunization. Sci. Rep..

[54] Lal H (2015). Efficacy of an adjuvanted herpes zoster subunit vaccine in older adults. N. Engl. J. Med.

[55] Cunningham AL (2016). Efficacy of the herpes zoster subunit vaccine in adults 70 years of age or older. N. Engl. J. Med.

[56] Gustafson CE, Kim C, Weyand CM, Goronzy JJ (2020). Influence of immune aging on vaccine responses. J. Allergy Clin. Immunol..

[57] McMahan K (2021). Correlates of protection against SARS-CoV-2 in rhesus macaques. Nature.

[58] Israelow B (2021). Adaptive immune determinants of viral clearance and protection in mouse models of SARS-CoV-2. Sci. Immunol..

[59] Corbett KS (2021). Immune correlates of protection by mRNA-1273 vaccine against SARS-CoV-2 in nonhuman primates. Science.

[60] Leist SR (2020). A mouse-adapted SARS-CoV-2 induces acute lung injury and mortality in standard laboratory mice. Cell.

[61] Israelow B (2020). Mouse model of SARS-CoV-2 reveals inflammatory role of type I interferon signaling. J. Exp. Med.

[62] Garcia-Beltran WF (2021). Multiple SARS-CoV-2 variants escape neutralization by vaccine-induced humoral immunity. Cell.

[63] Kuzmina A (2021). SARS-CoV-2 spike variants exhibit differential infectivity and neutralization resistance to convalescent or post-vaccination sera. Cell Host Microbe.

[64] Shen X (2021). Neutralization of SARS-CoV-2 Variants B.1.429 and B.1.351. N. Engl. J. Med.

[65] Tan AT (2021). Early induction of functional SARS-CoV-2-specific T cells associates with rapid viral clearance and mild disease in COVID-19 patients. Cell Rep..

[66] Mosca F (2008). Molecular and cellular signatures of human vaccine adjuvants. Proc. Natl Acad. Sci. USA.

[67] Cantisani R (2015). Vaccine adjuvant MF59 promotes retention of unprocessed antigen in lymph node macrophage compartments and follicular dendritic cells. J. Immunol..

[68] Didierlaurent AM (2017). Adjuvant system AS01: helping to overcome the challenges of modern vaccines. Expert Rev. Vaccines.

[69] De Giovanni M (2020). Spatiotemporal regulation of type I interferon expression determines the antiviral polarization of CD4(+) T cells. Nat. Immunol..

[70] Joyce MG (2022). A SARS-CoV-2 ferritin nanoparticle vaccine elicits protective immune responses in nonhuman primates. Sci. Transl. Med.

[71] Guo C (2021). A pathogen-like antigen-based vaccine confers immune protection against SARS-CoV-2 in non-human primates. Cell Rep. Med.

[72] Blom AG, Hilgers LA (2004). Sucrose fatty acid sulphate esters as novel vaccine adjuvants: effect of the chemical composition. Vaccine.

[73] Mohan T, Zhu W, Wang Y, Wang BZ (2018). Applications of chemokines as adjuvants for vaccine immunotherapy. Immunobiology.

[74] Wu K (2021). Variant SARS-CoV-2 mRNA vaccines confer broad neutralization as primary or booster series in mice. Vaccine.

