# Supplementary material for: Carbohydrate fatty acid monosulphate: oil-in-water adjuvant enhances SARS-CoV-2 RBD nanoparticle-induced immunogenicity and protection in mice
Source: NPJ Vaccines. 2023 Feb 14;8:18. doi: 10.1038/s41541-023-00610-4 (PMC9927065; doi:10.1038/s41541-023-00610-4)
Supplement: Supplementary file 1 — Supplementary Information [file 41541_2023_610_MOESM1_ESM.pdf]

Supplementary Material for

"Carbohydrate fatty acid monosulphate:oil-in-water adjuvant enhances SARS-CoV-2 RBD nanoparticle-induced immunogenicity and protection in mice"

Etsuro Nanishi[1, 2, 15], Francesco Borriello[1, 2, 3, 15, 18], Hyuk-Soo Seo[4, 5, 16], Timothy R. O'Meara[1, 16], Marisa E. McGrath[6, 16], Yoshine Saito[1], Jing Chen[7], Joann Diray-Arce[1, 2], Kijun Song[4], Andrew Z Xu[4], Soumik Barman[1, 2], Manisha Menon[1], Danica Dong[1], Timothy M. Caradonna[8], Jared Feldman[8], Blake M. Hauser[8], Aaron G. Schmidt[8, 9], Lindsey R. Baden[10], Robert K. Ernst[11], Carly Dillen[6], Jingyou Yu[12], Aiquan Chang[12], Luuk Hilgers[13], Peter Paul Platenburg[13], Sirano Dhe-Paganon[4, 5], Dan H. Barouch[12], Al Ozonoff[1, 2, 14], Ivan Zanoni[2, 3, 17], Matthew B. Frieman[6, 17], David J. Dowling[1, 2, 17], Ofer Levy[1, 2, 14, 17, *]

[1)] *Precision Vaccines Program*, Boston Children's Hospital, Boston, MA, USA.
[2)] Department of Pediatrics, Harvard Medical School, Boston, MA, USA.
[3)] Division of Immunology, Boston Children's Hospital, Boston, MA, USA.
[4)] Department of Cancer Biology, Dana-Farber Cancer Institute, Boston, MA, USA.
[5)] Department of Biological Chemistry and Molecular Pharmacology, Harvard Medical School, Boston, MA, USA.
[6)] Department of Microbiology and Immunology, Center for Pathogen Research, University of Maryland School of Medicine, Baltimore, MD, USA.
[7)] Research Computing Group, Boston Children's Hospital, Boston, MA, USA.
[8)] Ragon Institute of MGH, MIT, and Harvard, Cambridge, MA, USA.
[9)] Department of Microbiology, Harvard Medical School, Boston, MA, USA.
[10)] Department of Medicine, Brigham and Women's Hospital, Boston, MA, USA.
[11)] Department of Microbial Pathogenesis, University of Maryland School of Dentistry, Baltimore, MD, USA.
[12)] Center for Virology and Vaccine Research, Beth Israel Deaconess Medical Center, Harvard Medical School, Boston, MA, USA.
[13)] LiteVax B.V., Oss, The Netherlands.
[14)] Broad Institute of MIT & Harvard, Cambridge, MA, USA.
[15)] These authors contributed equally to this manuscript.
[16)] These authors contributed equally to this manuscript.
[17)] These authors contributed equally to this manuscript.
[18)] Present address: Generate Biomedicines, Cambridge, MA, USA.
* Corresponding author

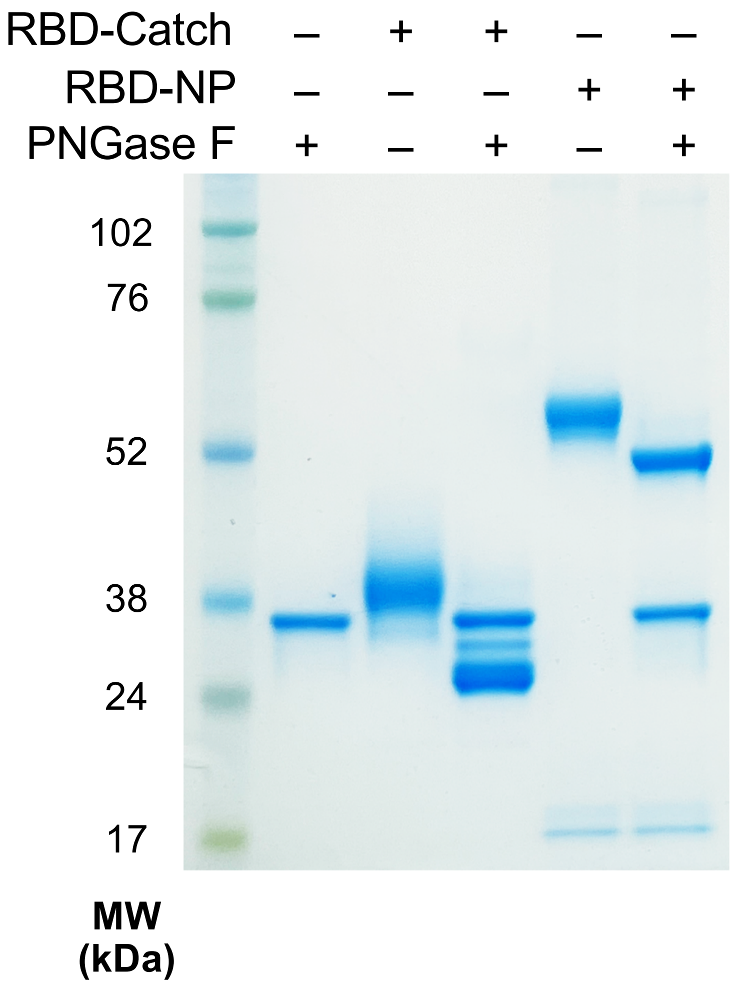

Supplementary Figure 1. RBD nanoparticle harbors multiple glycosylation patterns.
SDS-PAGE analysis of RBD expressing SpyCatcher (RBD-Catch) and RBD nanoparticle (RBD-NP) with or without Peptide-N-Glycosidase F (PNGase) treatment as described in Methods. Representative result of n=2 experiments is shown.

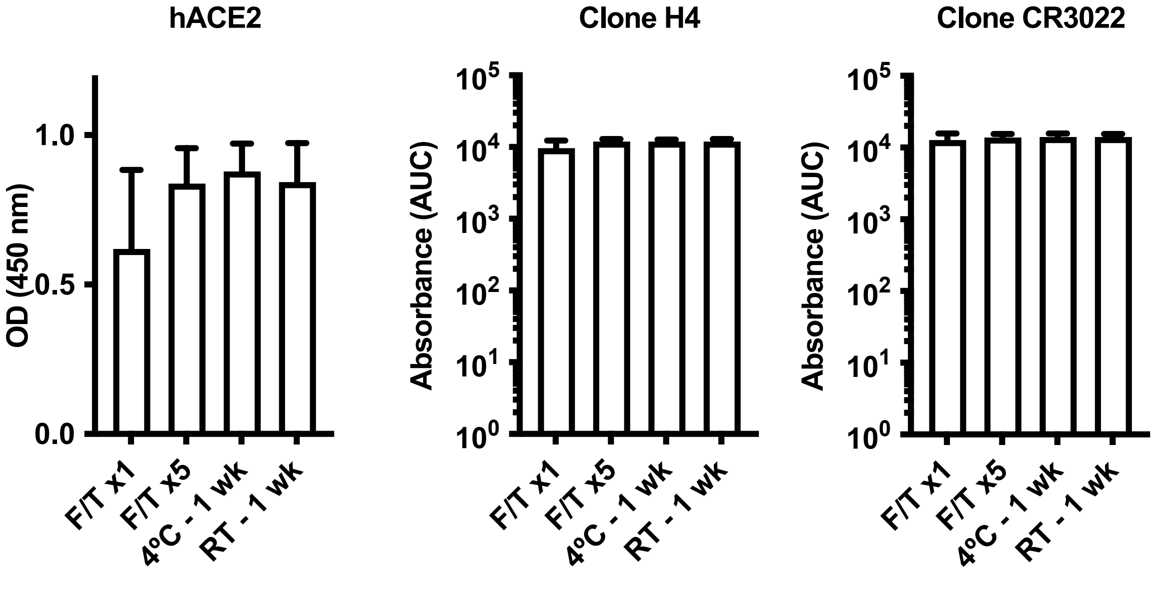

Supplementary Figure 2. RBD nanoparticle is stable under multiple storage conditions. ELISA plates were coated with RBD nanoparticles that underwent 1 (F/T x1) or 5 (F/T x5) freeze/thaw cycles or stored for 1 week at 4ºC (4ºC - 1wk) or room temperature (RT - 1wk). Binding of recombinant human ACE2 (hACE2) or anti-RBD H4 and CR3022 Ab clones was expressed as optical density (OD) at 450 nm or area under the curve (AUC). N = 4 experiments. Data are presented as mean and SD. Statistical significance was determined by one-way ANOVA corrected for multiple comparisons after Log-transformation of the raw data.

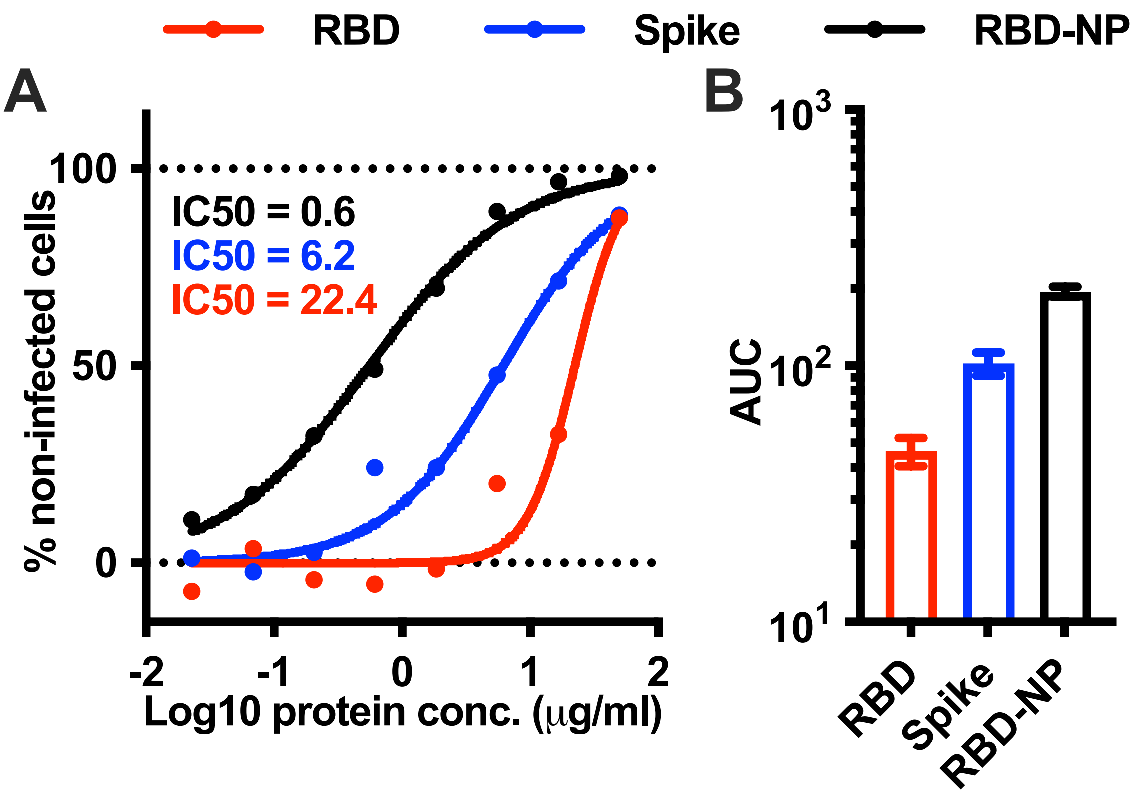

Supplementary Figure 3. RBD nanoparticle competes with SARS-CoV-2 *in vitro*.
(A, B) Vero cells were infected with SARS-CoV-2 in the presence or absence of RBD, Spike and RBD nanoparticles (RBD-NP) tested at multiple concentrations. Results are expressed as a percentage of non-infected cells. A non-linear curve was fitted for each protein and used to calculate IC50 (A) and area under the curve (B). Data are presented as mean and SD. N = 2 experiments.

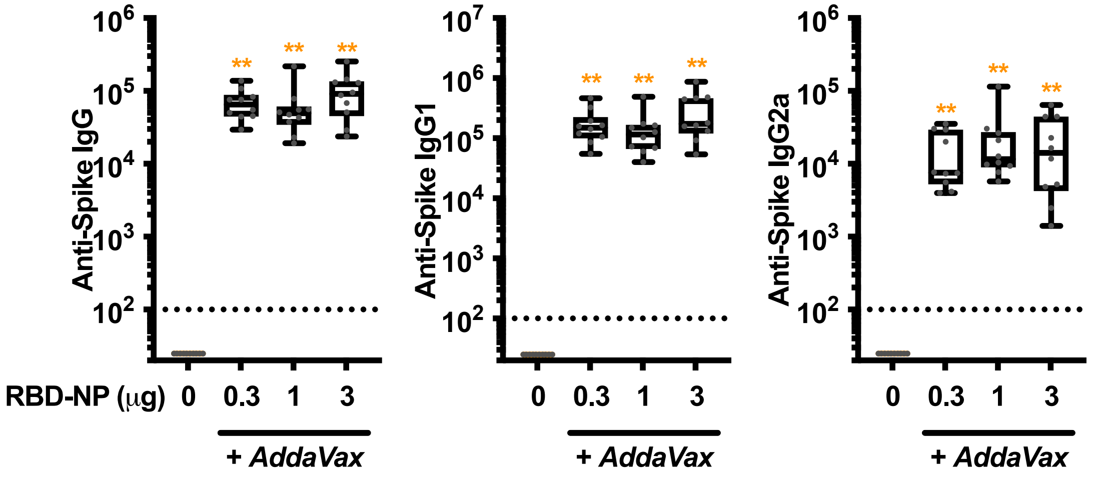

Supplementary Figure 4. Anti-RBD antibodies induced by RBD nanoparticle immunization recognize native RBD on Spike.

Anti-Spike IgG, IgG1 and IgG2a antibody titers were assessed in serum samples collected on Day 28 as indicated in Figure 2. Dotted lines indicate lower limit of detection. N = 10 mice per group. ** indicates $p \leq 0.01$. Statistical significance was determined by one-way ANOVA corrected for multiple comparisons after Log-transformation of the raw data. Box-and-whisker plots represent the minimum, first quartile, median, third quartile, and maximum value. Each symbol represents an individual mouse. The color code indicates comparisons among experimental groups.

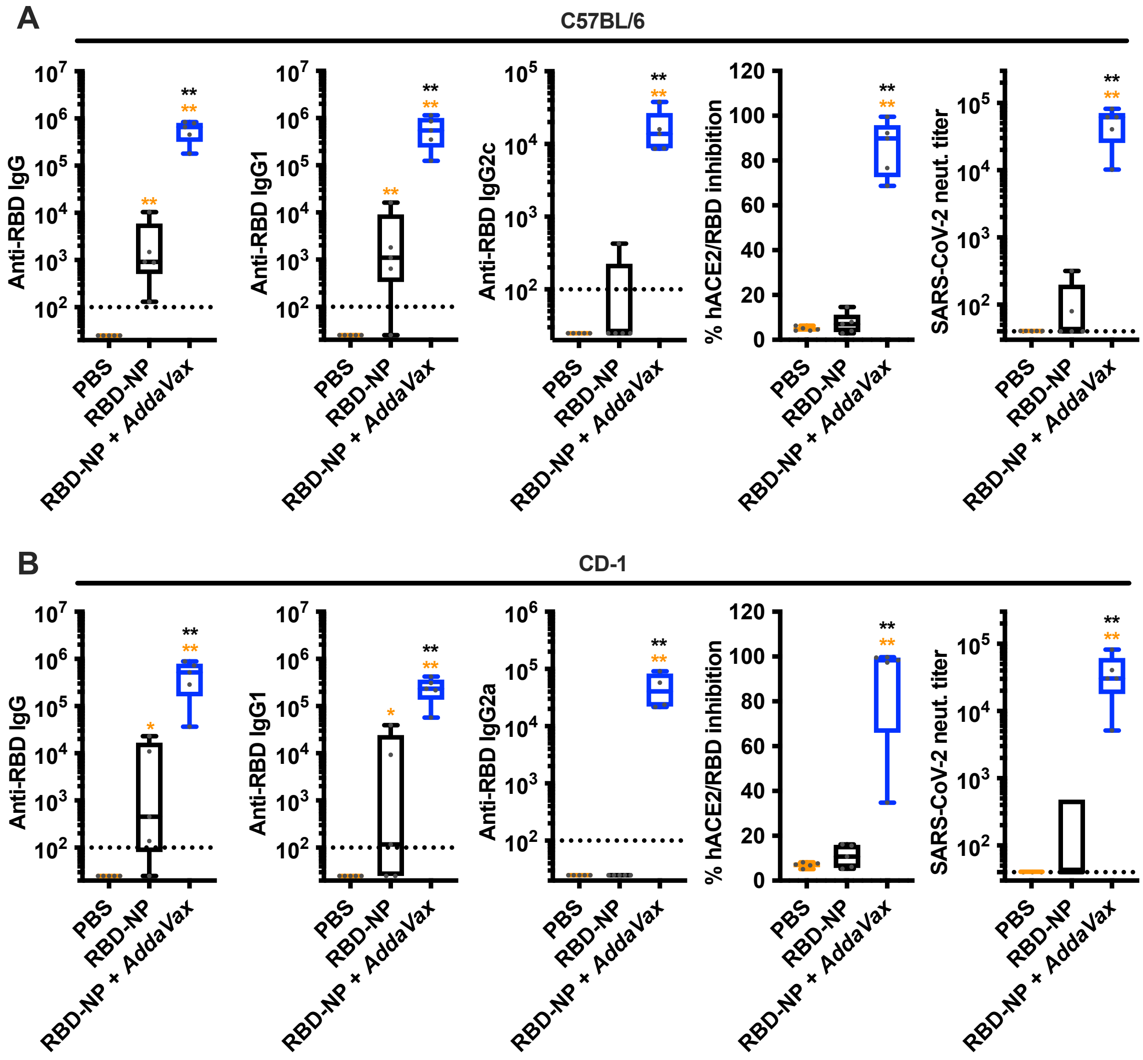

Supplementary Figure 5. RBD nanoparticle is immunogenic in multiple mouse strains. 3-month-old C57BL/6 (A) and CD-1 (B) mice were injected with PBS or immunized with 0.3 µg RBD nanoparticle (RBD-NP) alone or formulated with *AddaVax* on Day 0 (prime) and Day 14 (boost). Anti-RBD IgG, IgG1, and IgG2a antibody titers were measured in serum samples collected on Day 28. Dotted lines indicate a lower limit of detection. N = 5 mice per group. * and ** respectively indicate $p \leq 0.05$ and 0.01. Statistical significance was determined by one-way ANOVA corrected for multiple comparisons after Log-transformation of the raw data. Box-and-whisker plots represent the minimum, first quartile, median, third quartile, and maximum value. Each symbol represents an individual mouse. The color code indicates comparisons among experimental groups.

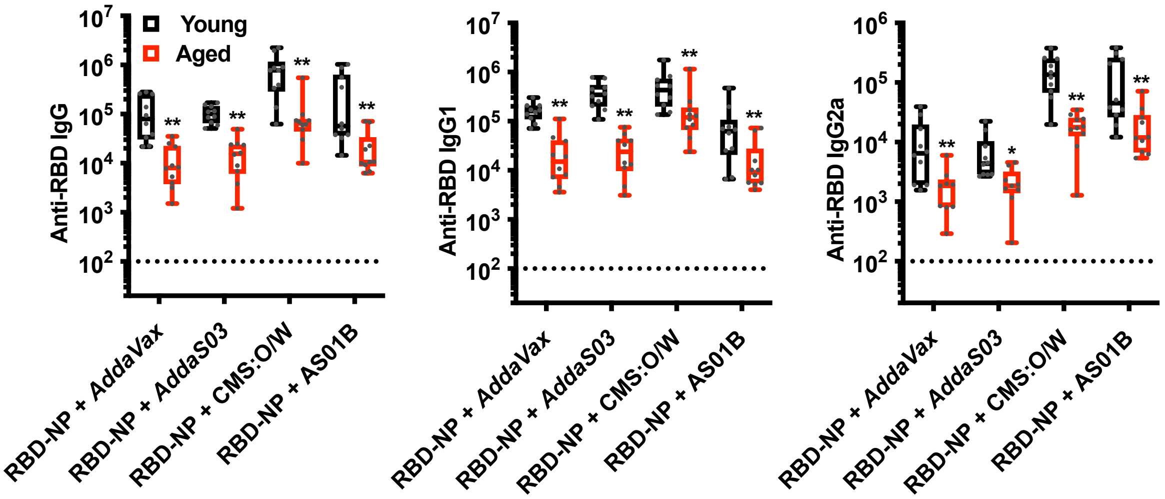

Supplementary Figure 6. Aged mice demonstrate reduced anti-RBD antibody response upon immunization.

Comparisons of anti-RBD IgG, IgG1, and IgG2a antibody titers between immunized young and aged mice as reported in Figure 4. N = 10 mice per group * and ** respectively indicate $p \leq 0.05$ and 0.01. Statistical significance was determined by two-way ANOVA corrected for multiple comparisons after Log-transformation of the raw data. Box-and-whisker plots represent the minimum, first quartile, median, third quartile, and maximum value. Each symbol represents an individual mouse. The color code indicates comparisons among experimental groups.

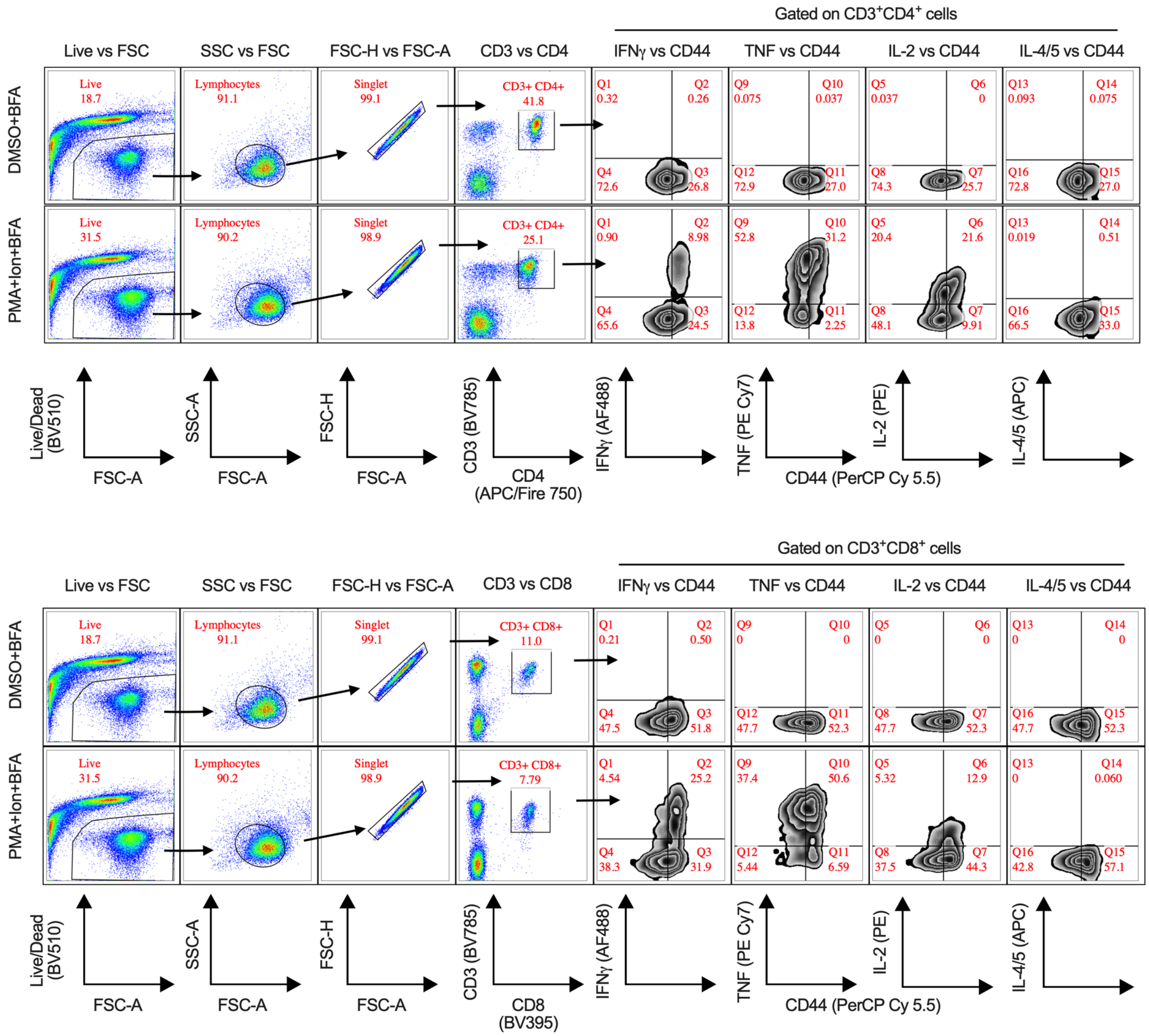

Supplementary Figure 7. Flow cytometry gating strategy.

Flow cytometry plots showing the gating strategy applied to identify RBD-specific CD4$^+$ and CD8$^+$ T cell responses after stimulating splenocytes with phorbol myristate acetate (PMA) and ionomycin.

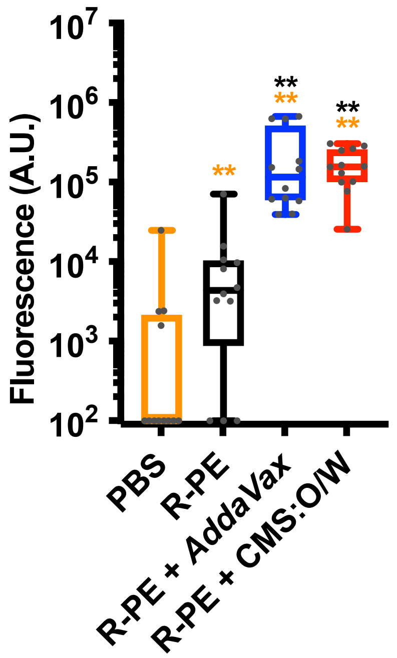

Supplementary Figure 8. *AddaVax* and CMS:O/W adjuvants promote antigen retention in the draining lymph node.
3-month-old BALB/c mice were injected intramuscularly with PBS, R-PE, and R-PE formulated with *AddaVax* or CMS:O/W adjuvant. 24 hours later, draining lymph nodes were collected, homogenized in water and fluorescence was measured in cleared supernatants. Results are expressed as arbitrary units (A.U.) of fluorescence. N = 12 mice per group. ** indicates $p \leq 0.01$. Statistical significance was determined by one-way ANOVA corrected for multiple comparisons after Log-transformation of the raw data. Box-and-whisker plots represent the minimum, first quartile, median, third quartile, and maximum value. Each symbol represents an individual mouse. The color code indicates comparisons among experimental groups.

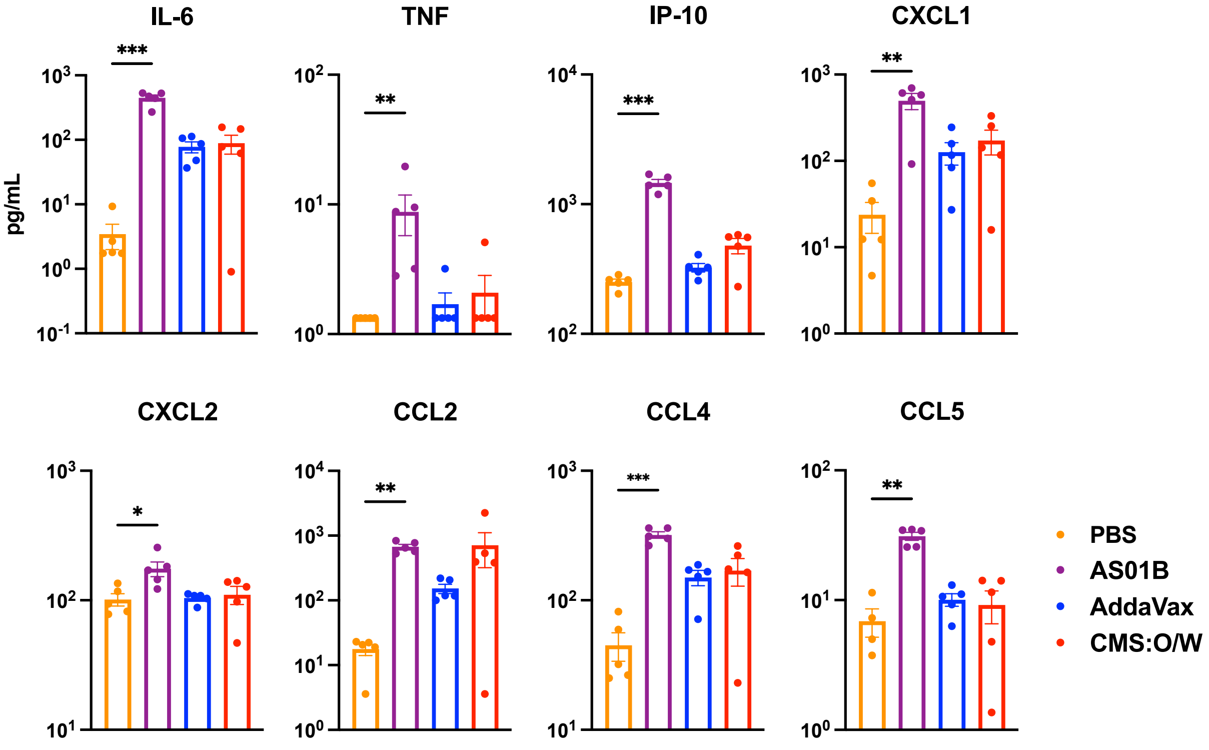

Supplementary Figure 9. CMS:O/W adjuvant demonstrates a favorable reactogenicity profile.
Young (3-month-old) BALB/c mice were injected with PBS, AS01B, AddaVax, or CMS:O/W. Serum samples were collected 24 hours later to assess cytokine and chemokine production by multiplexing bead array. Results are reported as mean ± SEM. Statistical significance was determined by Kruskal-Wallis test corrected for multiple comparisons. N = 4-5 mice per group. *, ** and *** respectively indicate $p < 0.05$, 0.01 and 0.001.
