# Supplementary material for: Carbohydrate fatty acid monosulphate: oil-in-water adjuvant enhances SARS-CoV-2 RBD nanoparticle-induced immunogenicity and protection in mice
Source: NPJ Vaccines. 2023 Feb 14;8:18. doi: 10.1038/s41541-023-00610-4 (PMC9927065; doi:10.1038/s41541-023-00610-4)
Supplement: Supplementary file 3 — REPORTING SUMMARY [file 41541_2023_610_MOESM3_ESM.pdf]

nature portfolio

Corresponding author(s): Ofer Levy

Last updated by author(s): Jan 11, 2023

Reporting Summary

Nature Portfolio wishes to improve the reproducibility of the work that we publish. This form provides structure for consistency and transparency in reporting. For further information on Nature Portfolio policies, see our Editorial Policies and the Editorial Policy Checklist.

Statistics

For all statistical analyses, confirm that the following items are present in the figure legend, table legend, main text, or Methods section.

n/a	Confirmed	
☐	☒	The exact sample size (*n*) for each experimental group/condition, given as a discrete number and unit of measurement
☐	☒	A statement on whether measurements were taken from distinct samples or whether the same sample was measured repeatedly
☐	☒	The statistical test(s) used AND whether they are one- or two-sided<br>*Only common tests should be described solely by name; describe more complex techniques in the Methods section.*
☒	☐	A description of all covariates tested
☐	☒	A description of any assumptions or corrections, such as tests of normality and adjustment for multiple comparisons
☐	☒	A full description of the statistical parameters including central tendency (e.g. means) or other basic estimates (e.g. regression coefficient) AND variation (e.g. standard deviation) or associated estimates of uncertainty (e.g. confidence intervals)
☐	☒	For null hypothesis testing, the test statistic (e.g. *F*, *t*, *r*) with confidence intervals, effect sizes, degrees of freedom and *P* value noted<br>*Give P values as exact values whenever suitable.*
☒	☐	For Bayesian analysis, information on the choice of priors and Markov chain Monte Carlo settings
☒	☐	For hierarchical and complex designs, identification of the appropriate level for tests and full reporting of outcomes
☒	☐	Estimates of effect sizes (e.g. Cohen's *d*, Pearson's *r*), indicating how they were calculated

Our web collection on statistics for biologists contains articles on many of the points above.

Software and code

Policy information about availability of computer code

Data collection	No unique software was used for data collection.
Data analysis	Data were analyzed with commercially available and open-source programs as stated in the methods section. Descriptive statistics were calculated with Prism 9.

For manuscripts utilizing custom algorithms or software that are central to the research but not yet described in published literature, software must be made available to editors and reviewers. We strongly encourage code deposition in a community repository (e.g. GitHub). See the Nature Portfolio guidelines for submitting code & software for further information.

Data

Policy information about availability of data

All manuscripts must include a data availability statement. This statement should provide the following information, where applicable:

- Accession codes, unique identifiers, or web links for publicly available datasets
- A description of any restrictions on data availability
- For clinical datasets or third party data, please ensure that the statement adheres to our policy

The authors declare that all data supporting the findings of this study are available within the supplementary information files.

Human research participants

Policy information about studies involving human research participants and Sex and Gender in Research.

Reporting on sex and gender	Peripheral blood was collected from human donor of both sex.
Population characteristics	Healthy adult study participants 18-40 years of age (n=8, 3 males and 5 females).
Recruitment	Healthy young donors were recruited via on line flier posted on Boston Children's Hospital internal website.
Ethics oversight	Boston Children's Hospital

Note that full information on the approval of the study protocol must also be provided in the manuscript.

Field-specific reporting

Please select the one below that is the best fit for your research. If you are not sure, read the appropriate sections before making your selection.

☒ Life sciences ☐ Behavioural & social sciences ☐ Ecological, evolutionary & environmental sciences

For a reference copy of the document with all sections, see nature.com/documents/nr-reporting-summary-flat.pdf

Life sciences study design

All studies must disclose on these points even when the disclosure is negative.

Sample size	Mouse experiments aimed to include in total 10 mice per group and were combined from two individual experiments. Sample size and age criteria were chosen empirically based on the results of previous studies.
Data exclusions	No data were excluded.
Replication	Data were combined from two individual experiments.
Randomization	Mice were randomly distributed in groups.
Blinding	Neutralization assay was performed by laboratory independent from the original laboratory. No other data was supplied until after the assay was complete. For the remaining assays, blinding was not preformed.

Reporting for specific materials, systems and methods

We require information from authors about some types of materials, experimental systems and methods used in many studies. Here, indicate whether each material, system or method listed is relevant to your study. If you are not sure if a list item applies to your research, read the appropriate section before selecting a response.

Materials & experimental systems

n/a	Involved in the study	
☐	☒	Antibodies
☐	☒	Eukaryotic cell lines
☒	☐	Palaeontology and archaeology
☐	☒	Animals and other organisms
☒	☐	Clinical data
☒	☐	Dual use research of concern

Methods

n/a	Involved in the study	
☒	☐	ChIP-seq
☐	☒	Flow cytometry
☒	☐	MRI-based neuroimaging

Antibodies

Antibodies used

anti-mouse CD44 [IM7, PerCP-Cy5.5, BioLegend #103032, 1:160]
anti-mouse CD3 [17A2, Brilliant Violet 785, BioLegend #100232, 1:40]
anti-mouse CD4 [RM4-5, APC/Fire 750, BioLegend 100568, 1:160]
anti-mouse CD8 [53-6.7, Brilliant UltraViolet 395, BD #563786, 1:80]
anti-mouse IFNγ [XMG1.2, Alexa Fluor 488, BioLegend #505813, 1:160]
anti-mouse TNF [MP6-XT22, PE Cy7, BioLegend # 506324, 1:160]

	anti-mouse IL-2 [JES6-5H4, PE, BioLegend # 503808, 1:40]<br>anti-mouse IL-4 [11B11, APC, BioLegend # 504106, 1:40]<br>anti-mouse IL-5 [TRFK5, APC, BioLegend # 504306, 1:40]<br>anti-mouse CD28 [1 µg/mL, BD, #553295]<br>anti-mouse CD49d [1 µg/mL, BD, #553313]
Validation	Commercial antibodies were used according to manufacturer's instructions and this is noted in the manuscript where appropriate.

Eukaryotic cell lines

Policy information about cell lines and Sex and Gender in Research

Cell line source(s)	THP1-Dual and THP1-Dual KO-MyD reporter cells were purchased from Invivogen. Human peripheral blood was collected from eight healthy adult study participants 18-40 years of age (3 males and 5 females).
Authentication	Reporter activity of the cell lines has been validated using functional assays.
Mycoplasma contamination	All cell lines were tested negative for mycoplasma.
Commonly misidentified lines (See ICLAC register)	Not applicable

Animals and other research organisms

Policy information about studies involving animals; ARRIVE guidelines recommended for reporting animal research, and Sex and Gender in Research

Laboratory animals	Female, 3-month-old BALB/c and C57BL/6J mice were purchased from Jackson Laboratory (Bar Harbor, ME), and CD-1 mice were purchased from Charles River Laboratories (Wilmington, MA). Female, 11-13 months old BALB/c mice purchased from Taconic Biosciences (Germantown, NY) were used for aged mice experiments. Female, 6-8 weeks old wild-type (#000664) and Myd88-/- (#009088) C57BL/6 were mice purchased from The Jackson Laboratory (Bar Harbor, ME).
Wild animals	Not applicable
Reporting on sex	All the experiments were performed with female mice.
Field-collected samples	Not applicable
Ethics oversight	Mice were housed under specific pathogen-free conditions at Boston Children's Hospital, and all procedures were approved under the Institutional Animal Care and Use Committee (IACUC) and operated under the supervision of the Department of Animal Resources at Children's Hospital (ARCH) (Protocol number 19-02-3897R). At the University of Maryland School of Medicine, mice were housed in a biosafety level 3 (BSL3) facility for all SARS-CoV-2 infections with all procedures approved under the IACUC (Protocol number #1120004).

Note that full information on the approval of the study protocol must also be provided in the manuscript.

Flow Cytometry

Plots

Confirm that:

☒ The axis labels state the marker and fluorochrome used (e.g. CD4-FITC).

☒ The axis scales are clearly visible. Include numbers along axes only for bottom left plot of group (a 'group' is an analysis of identical markers).

☒ All plots are contour plots with outliers or pseudocolor plots.

☒ A numerical value for number of cells or percentage (with statistics) is provided.

Methodology

Sample preparation	Mouse spleens were mechanically dissociated and filtered through a 70 µm cell strainer. After centrifugation, cells were treated with 1 mL ammonium-chloride-potassium lysis buffer for 2 min at RT. Cells were washed and plated in a 96-well U-bottom plate (2 x 106/well) and incubated overnight in RPMI 1640 supplemented with 10% heat-inactivated FBS, penicillin (100 U/ml), streptomycin (100 mg/ml), 2-mercaptoethanol (55 mM), non-essential amino acids (60 mM), HEPES (11 mM), and L-Glutamine (800 mM) (all Gibco).
Instrument	LSR II (BD) flow cytometer
Software	FlowJo v10.8.1 (FlowJo LLC)

Cell population abundance | No sorting was preformed.

Gating strategy | Gating strategies are supplied in supplemental figures.

☒ Tick this box to confirm that a figure exemplifying the gating strategy is provided in the Supplementary Information.
